# NCOR1 Orchestrates Transcriptional Landscapes and Effector Functions of CD4^+^ T Cells

**DOI:** 10.3389/fimmu.2020.00579

**Published:** 2020-04-03

**Authors:** Daniela Hainberger, Valentina Stolz, Ci Zhu, Michael Schuster, Lena Müller, Patricia Hamminger, Ramona Rica, Darina Waltenberger, Marlis Alteneder, Thomas Krausgruber, Anastasiya Hladik, Sylvia Knapp, Christoph Bock, Michael Trauner, Michael A. Farrar, Wilfried Ellmeier

**Affiliations:** ^1^Division of Immunobiology, Center for Pathophysiology, Infectiology and Immunology, Institute of Immunology, Medical University of Vienna, Vienna, Austria; ^2^Hans Popper Laboratory of Molecular Hepatology, Division of Gastroenterology and Hepatology, Department of Internal Medicine III, Medical University of Vienna, Vienna, Austria; ^3^CeMM Research Center for Molecular Medicine of the Austrian Academy of Sciences, Vienna, Austria; ^4^Laboratory of Infection Biology, Department of Internal Medicine I, Medical University Vienna, Vienna, Austria; ^5^Department of Laboratory Medicine, Medical University of Vienna, Vienna, Austria; ^6^Department of Laboratory Medicine and Pathology, Center for Immunology, Masonic Cancer Center, University of Minnesota, Minneapolis, MN, United States

**Keywords:** NCOR1, corepressor, CD4^+^ T cells, T helper differentiation, colitis, interferon γ, gene regulation

## Abstract

The differentiation of naïve CD4^+^ T cells into T helper (Th) subsets is key for a functional immune response and has to be tightly controlled by transcriptional and epigenetic processes. However, the function of cofactors that connect gene-specific transcription factors with repressive chromatin-modifying enzymes in Th cells is yet unknown. Here we demonstrate an essential role for nuclear receptor corepressor 1 (NCOR1) in regulating naïve CD4^+^ T cell and Th1/Th17 effector transcriptomes. Moreover, NCOR1 binds to a conserved *cis*-regulatory element within the *Ifng* locus and controls the extent of IFNγ expression in Th1 cells. Further, NCOR1 controls the survival of activated CD4^+^ T cells and Th1 cells *in vitro*, while Th17 cell survival was not affected in the absence of NCOR1. *In vivo*, effector functions were compromised since adoptive transfer of NCOR1-deficient CD4^+^ T cells resulted in attenuated colitis due to lower frequencies of IFNγ^+^ and IFNγ^+^IL-17A^+^ Th cells and overall reduced CD4^+^ T cell numbers. Collectively, our data demonstrate that the coregulator NCOR1 shapes transcriptional landscapes in CD4^+^ T cells and controls Th1/Th17 effector functions.

## Introduction

CD4^+^ T cells are key players of adaptive immunity. The generation of distinct effector T helper (Th) subsets upon activation of naïve CD4^+^ T cells is essential for a successful immune response against various pathogenic challenges. Th subsets are characterized by the expression of lineage-specific cytokines that contribute to the regulation of T cell-mediated immunity. However, Th cell differentiation has to be tightly controlled, since dysregulation can lead to immune-mediated diseases such as allergy, inflammation, or autoimmunity (1–3). Th lineage specification processes and effector functions are accompanied by the establishment and maintenance of lineage-specific gene expression programs that on top of key transcription factors are also regulated by epigenetic processes (4, 5). One important group of cofactors that connect repressive chromatin-modifying enzymes such as histone deacetylases (HDACs) with gene-specific transcription factors is formed by nuclear receptor corepressor 1 (NCOR1) and its related factor silencing mediator of retinoid and thyroid receptor (SMRT or NCOR2). NCOR1 was identified as a non-DNA-binding corepressor of thyroid hormone receptors and retinoic acid receptors and mediates transcriptional repression of nuclear receptors (NR) in the absence of their ligands (6). Subsequent studies demonstrated that NCOR1 interacts also with many other types of transcription factors (7–9), suggesting that NCOR1 functions as a transcriptional coregulator beyond NR-mediated control of gene expression. Germline deletion of NCOR1 results in embryonic lethality at E15.5 due to defects in CNS development and in definitive erythropoiesis (10). To investigate NCOR1 function in adult tissues and mice, conditional gene targeting approaches were performed as well as transgenic mouse models expressing mutant versions of NCOR1 were generated. These approaches demonstrated a key role for NCOR1 in the control of muscle physiology (11), in the regulation of adipocyte function (12, 13), in liver metabolism (14), in macrophage-mediated repression of atherosclerosis (15), and in dendritic cell-mediated regulation of immune tolerance (16). Collectively, these studies indicate that NCOR1 is an essential tissue-specific regulator of gene expression in mammals during development, differentiation, cell homeostasis, and metabolism (8).

The role of NCOR1 in T cells is largely unknown. Studies with NCOR1 knockout mice suggested an essential role for NCOR1 during early thymocyte development, since NCOR1-deficient fetal thymocytes displayed a developmental block at the double-negative (DN) stage in fetal thymic organ cultures (10). Recently, a conditional gene targeting approach was used to generate mice with a T cell-specific deletion of NCOR1 to analyze the role of NCOR1 in adult thymopoiesis (17, 18). Leng and colleagues showed that NCOR1 is part of the transcriptional complexes that repress the pro-apoptotic factor BIM in activated thymocytes, thereby promoting thymocyte survival during positive selection (18). Our laboratory demonstrated that NCOR1 promotes also the survival of single-positive (SP) thymocytes, since *Cd4*-Cre-mediated loss of NCOR1 resulted in reduced SP thymocyte numbers (17). As a consequence of the important role of NCOR1 for thymocyte survival, peripheral T cell numbers are reduced in the absence of NCOR1, indicating that NCOR1 is essential for the generation of the peripheral T cell pool (17, 18). However, whether NCOR1 regulates peripheral T cell activation, differentiation, and effector functions has not been investigated.

Here we studied, by analyzing mice with a T cell-specific deletion of NCOR1, the impact of NCOR1 deletion on CD4^+^ T cell activation and Th1/Th17 effector functions. By combining ChIP-seq and RNA-seq approaches, we identified NCOR1 target genes and showed that NCOR1 is an important regulator of transcriptional landscapes in naïve and effector CD4^+^ T cells. Further, we assessed cell proliferation and cell survival of *in vitro*-activated and polarized NCOR1-deficient CD4^+^ T cells. In addition, we employed an adoptive transfer model of colitis to study the impact of NCOR1 deletion during CD4^+^ T cell-mediated autoinflammation. Together, our study demonstrates a novel role for the transcriptional cofactor NCOR1 in regulating transcriptional programs and effector functions in CD4^+^ T cells.

## Materials and Methods

### Animal Models

Animal experiments were evaluated by the ethics committees of the Medical University of Vienna and approved by the Austrian Federal Ministry for Education, Science and Research (GZ:BMWF-66.009/0105-WF/II/3b/2014, BMBWF-66.009/0039-V/3b/2019). Animals were maintained in research facilities of the Department for Biomedical Research at the Medical University of Vienna. Animal husbandry and experiments were performed under national laws in agreement with guidelines of the Federation of European Laboratory Animal Science Associations (FELASA), which correspond to Animal Research: Reporting of *in vivo* Experiments from the National Center for the Replacement, Refinement and Reduction of Animals in Research (ARRIVE) guidelines. *Ncor1*^f/f^ mice (11) were kindly provided by Johan Auwerx. *Cd4*-Cre mice were kindly provided by Chris Wilson. OT-II TCR transgenic mice (19) were kindly provided by Dagmar Stoiber-Sakaguchi. NCOR1 RID mice were obtained from Jackson laboratory. CD45.1 and *Rag2*^−/−^ mice were kindly provided by Jochen Hühn. *Ncor1*^f/f^, *Cd4*-Cre (NCOR1 cKO^Cd4^) and *Ncor1*^f/f^ OTII-tg, *Cd4*-Cre (OT-II, NCOR1 cKO^Cd4^) were previously described (17). Analyzed mice were 8–12 weeks of age and of mixed sex unless otherwise stated.

### Antibodies and Cytokines

The antibodies and cytokines used in this study are listed in Supplementary Table 2.

### Purification of Naïve CD4^+^ T Cells

Spleen and lymph nodes were isolated and single cell suspensions were made using a 70 μm cell strainer (Corning) in a 6-well-plate (Sarstedt) containing PBS (Sigma) with 2% fetal bovine serum (FBS) (Sigma/biowest) (PBS/FBS). Red blood cells were removed using BD Pharm Lyse™ (BD Biosciences). Cells were resuspended in PBS/FBS containing a mastermix of biotinylated antibodies (Gr-1, B220, NK1.1, CD11b, CD11c, CD8α, and TER-119). CD4^+^ T cells were enriched by negative depletion using magnetic streptavidin beads (MagniSort SAV Negative Selection beads; Invitrogen) according to the manufacturer's instructions. Depleted cells were further sorted into naïve CD4^+^ T cells (CD25^−^CD44^lo^CD62L^+^) on a BD FACSAria™ II (BD Biosciences) or on a SH800 (SONY).

### CD4^+^ T Cell Activation, Differentiation, and Cell Proliferation Analysis *in vitro*

For Th0 conditions, sorted naïve CD4^+^ T cells were stimulated (day 0) with plate-bound anti-CD3ε (1 μg/ml; BD Biosciences) and anti-CD28 (3 μg/ml; BD Biosciences) on 48-well-plates (0.3 × 10^6^ cells/well) in 1 ml T cell medium/well (RPMI1640 supplemented with 10% FBS [Sigma/biowest], antibiotics, 50 mM β-mercaptoethanol) supplemented with 20 U/ml recombinant human IL-2 (rhIL-2) (Peprotech) for 3 days, unless otherwise stated. For the assessment of cell proliferation, 1–10 × 10^6^ naïve CD4^+^ T cells were labeled using Cell Proliferation Dye eFluor™ 450 (Thermo Scientific) according to the manufacturer's protocol prior to activation. Th1 and Th17 cells were generated from sorted naïve CD4^+^ T cells activated with anti-CD3ε/anti-CD28 for 3 days in the presence of 20 U/ml rhIL-2 (Peprotech), 5 ng/ml IL-12 (R&D Systems) and 3 μg/ml anti-IL-4 (BioXcell) for Th1 cells, and in the presence of 2 ng/ml TGFβ, 10 μg/ml IL-6, 10 μg/ml IL-1α, and 10 μg/ml IL-1β for Th17 cells. For cytokine detection, activated cells were restimulated for 4 h with phorbol 12-myristate 13-acetate (PMA, 25 ng/ml) and ionomycin (Iono, 750 ng/ml) (both from Sigma-Aldrich) in the presence of GolgiStop (BD Biosciences).

### Extracellular and Intracellular Staining

*Ex vivo* isolated T cells and *in vitro*-cultured CD4^+^ T cells were incubated with Fc-block (1:250; BD Biosciences) followed by surface staining. Dead cells were excluded using Fixable Viability Dye eFluor® 506 or 780 (Thermo Scientific) according to the manufacturer's protocol. For intracellular cytokine stainings, cells were fixed with Cytofix Fixation Buffer (BD Biosciences), permeabilized with Perm/Wash Buffer (BD Biosciences) and stained according to the manufacturer's protocol. For intracellular transcription factor stainings, cells were fixed and permeabilized using the Foxp3 Staining Buffer Set (Thermo Scientific) according to the manufacturer's protocol and stained with the appropriate antibodies. Cells were measured with a BD LSRFortessa™ (BD Biosciences) and analyzed using FlowJo v10.2 software (TreeStar).

### *In vivo* CD4^+^ T Cell Proliferation Assays

MACS-purified CD4^+^ T cells from OT-II, WT and OT-II, NCOR1 cKO^Cd4^ mice were labeled with Cell Trace™ Cell proliferation dye (Thermo Fisher Scientific) according to the manufacturer's protocol. Cell tracker dye-labeled cells (0.5 × 10^6^ cells/200 μl) were injected i.v. into CD45.1^+^ mice. On the next day mice were immunized into the footpad with 50 μl ovalbumin (OVA)-peptide/CFA emulsion (50% incomplete Freund's adjuvant (Sigma) supplemented with 10 mg/ml of heat-killed Mycobacterium tuberculosis (strain H37Ra; Difco) and 50% of 2 mg/ml OVA-peptide (amino acids 323-339; Sigma Aldrich) resuspended in PBS). The dLNs were harvested 3 days later. Single cell suspensions were stained with extracellular antibodies. Cells were acquired on a BD LSRFortessa™ (BD Biosciences) and analyzed using FlowJo v10.2 software (TreeStar).

### Apoptosis Detection Assay

Activated CD4^+^ T cells were harvested at different times (0, 12, 24, and 72 h) and extracellular staining was performed as described above. The various stages of apoptotic cells were assessed using the eBioscience™ Annexin V detection Kit eFluor™ 450 (Thermo Fisher Scientific) and eBioscience™ 7-AAD Viability Staining Solution (Thermo Fisher Scientific) according to the manufacturer's protocol.

### Immunization of OTII-Transgenic Mice

OT-II, WT and OT-II, NCOR1 cKO^Cd4^ mice were s.c. immunized with OVA-peptide/CFA emulsion. The dLNs were isolated on day 3 and single cell suspensions were seeded into a 48-well-plate (4 × 10^6^ cells in 500 μl T cell medium). On the next day the cells were either activated with PMA/Iono as described above or restimulated with OVA-peptide and Golgi Stop (BD Biosciences) for 6 h. Extracellular and intracellular stainings were performed as described above.

### Lamina Propria (LP) Cell and Intraepithelial Lymphocyte (IEL) Isolation

Small intestines (SIs) were isolated and transferred into petri dishes with HBSS on ice. The stool was removed and SIs were cut longitudinally into small pieces. Tissue fragments were transferred into a tube with 30 ml wash solution [1 X HBSS, HEPES-bicarbonate (pH 7.2) and 2% FBS] and vortexed for 15 s to remove the mucus. Further tissue fragments were purified via filtering through a 100 μm cell strainer and the tissues remaining in the filters were transferred into a new tube. Washing steps were repeated two more times. Subsequently, intestinal tissues were transferred in a new petri dish with HBSS and cut into very tiny pieces. The cut tissue fragments were put in a new tube and incubated with EDTA solution (10% FBS, 1 X HBSS, 15 mM HEPES, 1 mM EDTA, pH 7.2) at 37°C for 15 min while shaking at 200 rpm. Afterwards, the solution was passed through a 100 μm filter and the IEL-containing flow-through was washed with RPMI/10% FBS and centrifuged at 600 g for 7 min. The cell pellet was resuspended in collagenase D solution (RPMI1640 supplemented with 1 mM MgCl_2_, 1 mM CaCl_2_, 5% FBS, and 100 units/ml collagenase D (Gibco™, Thermo Fisher Scientific) and incubated at 37°C for 30 min while shaking at 200 rpm. The remaining tissue pieces of the EDTA incubation step were washed with RPMI/10% FCS and digested with collagenase D solution for 1 h at 37°C while shaking at 200 rpm to isolate lamina propria cells. After collagenase D digestion, IELs or LP cells were resuspended in DMEM containing 40% Percoll (GE Healthcare), overlayed with DMEM/80% Percoll and centrifuged at room temperature for 30 min at 2,000 rpm at low acceleration/deceleration settings. Cells from the gradient interface were collected, washed and resuspended in PBS/FBS.

### Adoptive CD4^+^ T Cell Transfer Model of Colitis and Analysis of Tissues

0.5 × 10^6^ naïve WT and NCOR1 cKO^Cd4^ CD4^+^ T cells were i.p. injected into Rag2^−/−^ mice. Recipient mice were analyzed after 8 weeks. Spleens, mLNs, SI-LP cells, and SI-IELs were isolated and cells were analyzed by extracellular or intracellular stainings as described above. For histological analysis, swiss rolls were prepared from coli of diseased mice as described (20).

### Histology and Multicolor Immunofluorescence Microscopy

Fixed tissue samples were preceded with a tissue processor (Thermo Fisher Scientific). For hematoxylin and eosin (H&E) stainings, histologic evaluation was performed on 5 μm thick sections and stained with hematoxylin and eosin. High power field images (i.e. 400x magnification) were collected from each colon tissue. For immunofluorescence staining, tissue sections were heated with the antigen retrieval buffer (citrate buffer, pH 6) to 95°C and blocked with 10% goat serum in PBS. Sections were subsequently incubated with rabbit anti-CD3 and bound antibodies were visualized with Alexa Fluor 488 anti-rabbit. Nuclei were stained with 4′,6-diamidino-2-phenylindole (DAPI). Immunostained sections were scanned using an Olympus fluorescence microscope. Four representative high power field images were captured from each colon and processed using Olympus cellSens software.

### RNA Sample Preparation and RNA Sequencing

CD4^+^ T cells were isolated from spleen and lymph nodes and naïve CD4^+^ T cells (CD25^−^CD44^lo^CD62L^+^) were sorted or polarized into Th1/Th17 lineage as described above. RNA from 3 × 10^6^ cells was isolated with QIAshredder, RNaeasy Mini Kit, and RNase-Free DNase Set (Qiagen) according to manufacturer's protocol. Three biological replicates were generated for each genotype and T cell subset. The amount of RNA was estimated with Qubit 2.0 Fluorometer (Thermo Fisher Scientific) and the RNA integrity number with Experion Automated Electrophoresis System (Bio-Rad). RNA-seq libraries were prepared. The libraries were sequenced by the Biomedical Sequencing Facility at CeMM using the Illumina HiSeq 3000/4000 platform and the 50 bp single-read configuration. Final RNA-seq data included infinite fold change (FC) values, which were excluded for the generation of volcano plots. Venn diagrams were generated with the Venny 2.1 online tool (21).

### ChIP Sequencing Sample Preparation

Naïve CD4^+^ T cells (CD25^−^CD44^lo^CD62L^+^) were isolated as described above and 3 × 10^6^ cells were used for each of the two biological replicates. ChIP was performed using anti-NCOR1 (Cell Signaling Technology; #5948S). Cells were processed according to the ChIPmentation protocol (22). DNA amount was measured with Qubit 2.0 Fluorometer (Thermo Fisher Scientific) and the DNA integrity with a Bioanalyzer (Agilent). The libraries were sequenced by the Biomedical Sequencing Facility at CeMM using the Illumina HiSeq 3000/4000 platform and the 50 bp single-read configuration.

### ChIP Sequencing Analysis

The sequencing data in base call (BCL) format were converted via custom software based on Picard tools in a workflow relying on unaligned BAM files. NGS adapter sequences annotated by Picard tools via BAM XT tags were hard masked during FASTQ format conversion (Picard SamToFastq), before NGS reads were aligned with the Bowtie2 short read aligner (23) (version 2.2.5) to the GRCm38 (UCSC mm10) reference genome assembly (24) in end-to-end mode. The resulting alignment files were post-processed, including propagation of raw data annotation (Picard MergeSamAlignment). Peaks were characterized with the MACS2 callpeak algorithm (25) (version 2.1.2) following factor-specific protocols provided by the developers (26). The MACS2 bdgcmp algorithm was used in Poisson *P*-value with Benjamini-Hochberg false discovery rate (qpois), log fold-enrichment (logFE) and subtraction modes to generate corresponding signal plots linked into a UCSC Genome Browser track hub for visualization of the data set. For samples with two or more replicates, a differential binding analysis was carried out utilizing the Bioconductor DiffBind package (27) (version 2.12.0). Based on peak regions previously defined by MACS2 for each sample, DiffBind compiled a consensus peak set and tested for significantly differentially occupied peaks by means of the Bioconductor DESeq2 package. A set of contrasts was extracted from the generalized linear model and the resulting peak lists were annotated with the Bioconductor ChIPpeakAnno package (28) (version 3.18.2). Based on genome sequence regions underlying the consensus peak set, *de novo* motifs were characterized via the Bioconductor rGADEM package (29).

### Statistical Analysis

No statistical methods were used to predetermine the sample size. The data shown indicate the mean. The number of independent experiments for each data set requiring a statistical analysis is provided in each figure legend. The statistical analyses were performed using Prism 8 Software (GraphPad Inc). As indicated in each figure legend, *P*-values were calculated with an unpaired two-tailed *t*-test, paired *t*-test (a normal distribution of data points was assumed) or with one-way ANOVA. No data were excluded and no specific randomization of animals or blinding of investigators was applied.

## Results

### NCOR1 Regulates Transcriptional Landscapes in Naïve CD4^+^ T Cells

**Figure 1:**
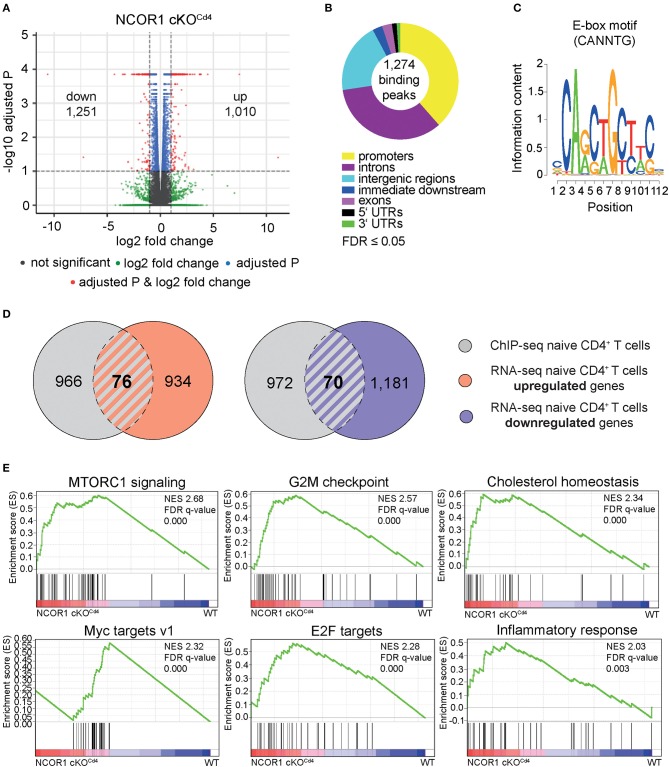
NCOR1-dependent transcriptional profile and NCOR1 target genes in naïve CD4^+^ T cells. **(A)** RNA was isolated from naïve WT and NCOR1 cKO^Cd4^ CD4^+^ T cells and subjected to RNA sequencing. Three independent WT and NCOR1 cKO^Cd4^ CD4^+^ T cells batches were prepared on the same day. Volcano plot depicts a comparison of global gene expression profiles between naïve WT and NCOR1 cKO^Cd4^ CD4^+^ T cells. The x-axis displays fold change (log_2_) and the y-axis indicates *P*-values (–log_10_). 1,010 and 1,251 genes were up- and downregulated in NCOR1 cKO^Cd4^ CD4^+^ T cells (FDR ≤ 0.05 was used as selection criteria). **(B)** NCOR1 ChIP-sequencing of naïve WT CD4^+^ T cells revealed 1,274 significant NCOR1 binding peaks. Two independent batches were sequenced. The donut chart shows the distribution of NCOR1 binding peaks across the genome of naïve WT CD4^+^ T cells (promoters, introns, intergenic regions, immediate downstream, exons, 5′ untranslated regions (UTRs), and 3′ UTRs). The 1,274 binding peaks mapped to 1,042 genes, and this gene list was used for further analysis. **(C)** Sequence shows DNA-binding motif enriched within NCOR1 recruitment sites as revealed with the Bioconductor rGADEM package. **(D)** Venn diagrams showing an overlay of NCOR1-bound genes (gray) with genes that are up- (left Venn diagram) and downregulated (right Venn diagram) in NCOR1-deficient naïve CD4^+^ T cells. **(E)** Gene set enrichment analysis (GSEA) plots generated from RNA-seq expression data of WT and NCOR1 cKO^Cd4^ naïve CD4^+^ T cells. Gene sets of the hallmark gene set collection of the Molecular Signature Database (MSigDB) were used. The top 6 enriched hallmark gene sets are shown. A detailed list of differentially expressed genes within these hallmark gene sets is provided in Supplementary Figure 2A. The bar codes indicate the location of the members of the gene set in the ranked list of all genes. FDR, false discovery rate; NES, normalized enrichment score.

Previous studies have shown that NCOR1 regulates the survival of thymocytes during negative and positive selection and that as a consequence a T cell-specific loss of NCOR1 leads to a 2-fold reduction of peripheral T cell numbers (17, 18). These results indicate that NCOR1 is also crucial for the generation of the peripheral T cell pool. To investigate whether NCOR1 is required for the maintenance of CD4^+^ T cell homeostasis and function, we performed a genome-wide transcriptome analysis using RNA sequencing (RNA-seq) approaches and compared the transcriptional profiles of WT and *Ncor1*^f/f^, *Cd4*-Cre (designated as NCOR1 cKO^Cd4^ throughout the manuscript) naïve CD4^+^ T cells (CD25^−^CD44^lo^CD62L^+^). This revealed a dysregulation of 2,261 genes, with 1,010 genes upregulated, and 1,251 genes downregulated (FDR ≤ 0.05 was used as selection criteria) in the absence of NCOR1 (Figure 1A). To identify potential NCOR1 target genes, we mapped the genome-wide distribution of NCOR1 binding sites in naïve CD4^+^ T cells using chromatin immunoprecipitation followed by high-throughput sequencing approaches (ChIP-seq). Using this experimental approach we identified genome-wide 1,274 NCOR1 binding peaks in naïve CD4^+^ T cells, with a large fraction of binding sites in promoters, introns and intergenic regions (Figure 1B, Supplementary Figure 1). These 1,274 binding peaks map to 1,042 gene loci, which were used for further analysis. Moreover, a *de novo* motif search revealed an enrichment of an E-box motif (30) (CANNTG) (Figure 1C). Analysis of the 1,042 NCOR1 bound gene loci with the 1,010 genes upregulated in NCOR1 cKO^Cd4^ T cells revealed an overlap of 76 genes, suggesting a direct repression of these genes by NCOR1 (Figure 1D, left Venn diagram; Supplementary Table 1). Of note, from the 1,251 genes downregulated in the absence of NCOR1, 70 gene loci contained NCOR1 binding sites (Figure 1D, right Venn diagram; Supplementary Table 1). Further, to identify the impact of the transcriptional changes on cellular processes and function, we performed a gene set enrichment analysis (GSEA) (31–33). This revealed enrichment of the hallmark gene sets “MTORC1 signaling,” “G2M checkpoint,” “cholesterol homeostasis,” “Myc targets v1,” “E2F targets,” and “inflammatory response” in NCOR1 cKO^Cd4^ naïve CD4^+^ T cells (Figure 1E, Supplementary Figure 2A). Together, these data indicate an essential role for NCOR1 in setting up transcriptional programs in naïve CD4^+^ T cells.

### Reduced Survival of Naïve NCOR1-Deficient CD4^+^ T Cells Upon Activation

**Figure 2:**
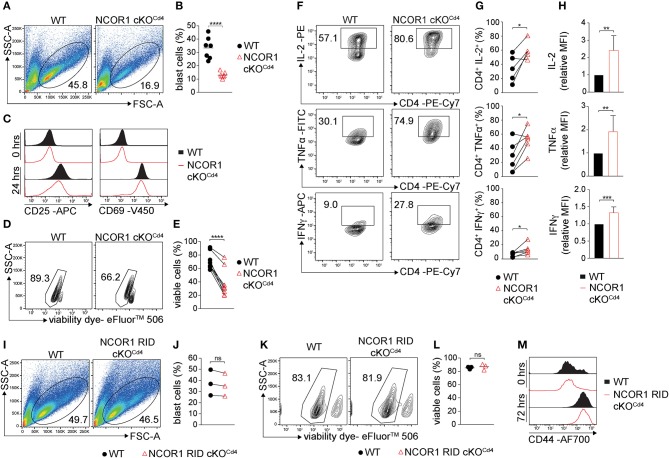
NCOR1 controls the survival of *in vitro*-activated CD4^+^ T cells. **(A)** Naïve WT and NCOR1 cKO^Cd4^ CD4^+^ T cells were activated with anti-CD3ε/anti-CD28 and cultured in the presence of recombinant hIL-2 for 3 days (= Th0 condition). Pseudocolor plots show side scatter (SSC-A) vs. forward scatter (FSC-A). Regions indicate T cell blasts. **(B)** Summary diagram shows the percentage of T cell blasts from all experiments performed as described in **(A)**. **(C)** Representative histograms depict expression of CD25 (left) and CD69 (right) on WT and NCOR1 cKO^Cd4^ Th0 cells before (0 h; upper histograms) and 24 h (lower histograms) after activation. **(D)** Representative contour plots of viability dye vs. side scatter (SSC-A) for WT and NCOR1 cKO^Cd4^ Th0 cells. **(E)** Summary of all experiments performed as described in **(D)** shows percentages of viable WT and NCOR1 cKO^Cd4^ Th0 cells. **(F)** Contour plots depict IL-2, TNFα, and IFNγ vs. CD4 expression in WT and NCOR1 cKO^Cd4^ Th0 cells. **(G)** Summary showing results of all experiments as described in **(F)**. **(H)** Summary graphs depicting cytokine expression levels as mean fluorescence intensity (MFI). For each experiment, WT levels were set as 1 and relative MFI of IL-2, TNFα, and IFNγ are shown. **(I)** MACS-purified naïve WT and NCOR1 RID cKO^Cd4^ CD4^+^ T cells were activated as described in **(A)**. Pseudocolor plots show side scatter (SSC-A) vs. forward scatter (FSC-A) for WT and NCOR1 RID cKO^Cd4^ Th0 cells. **(J)** Summary diagrams of all experiments performed as described in **(I)** showing the percentages of WT and NCOR1 RID cKO^Cd4^ Th0 blast cells. **(K)** Contour plots of viable WT and NCOR1 RID cKO^Cd4^ Th0 cells. **(L)** Percentages of viable WT and NCOR1 RID cKO^Cd4^ Th0 cells from all experiments as described in **(K)**. **(M)** Representative histograms depict expression of CD44 on WT and NCOR1 RID cKO^Cd4^ Th0 cells before (0 h, upper histograms) and 72 h (lower histograms) after activation. **(B,L)** The horizontal bars indicate the mean. *P < 0.05, **P < 0.01, ***P < 0.001, ****P < 0.0001, ns, not significant (unpaired two-tailed *t*-test or paired *t*-test for **E,G,J**). **(A,D,F,I,K)** Numbers indicate the percentage of cells in the respective regions. **(E,G,J)** Lines indicate paired experiments. Data are representative **(A,C,D,F,I,K,M)** or show summary **(B,E,G,H,J,L)** of 7 **(B)**, 3 **(J,L)**, 8 **(E)**, and 5–6 **(G,H)** samples that were analyzed in 3–8 independent experiments.

We next determined the impact of the transcriptional changes and the dysregulation of various cellular processes in the absence of NCOR1 on TCR-mediated activation of NCOR1 cKO^Cd4^ CD4^+^ T cells. Upon anti-CD3ε/anti-CD28 stimulation, WT CD4^+^ T cells formed typical lymphoblasts after 3 days, while the frequency of NCOR1-deficient CD4^+^ T cell lymphoblasts was severely reduced (Figures 2A,B). Of note, NCOR1-deficient CD4^+^ T cell blasts did not escape *Ncor1* deletion (Supplementary Figure 3A). CD25 and CD69, two early activation markers, were both upregulated to a similar degree in WT and NCOR1 cKO^Cd4^ CD4^+^ T cells on day 1 of T cell stimulation (Figure 2C), suggesting that the reduction in T cell blasts in the absence of NCOR1 was not due to early TCR signaling defects. However, the percentage of viable *in vitro*-activated NCOR1 cKO^Cd4^ CD4^+^ T cells on day 3 was significantly reduced in comparison to *in vitro*-activated WT CD4^+^ T cells, suggesting a diminished survival rate of NCOR1-deficient T cells upon activation (Figures 2D,E). To obtain insight into the kinetics of apoptosis induction in NCOR1-deficient CD4^+^ T cells, we activated WT and NCOR1 cKO^Cd4^ CD4^+^ T cells for 12, 24, and 72 h with anti-CD3ε/anti-CD28 and assessed cell survival by performing Annexin V and 7-AAD stainings (Supplementary Figure 3B). Already 12 h after activation, the fraction of viable (i.e., AnnexinV^−^/7-AAD^−^) NCOR1 cKO^Cd4^ CD4^+^ T cells was reduced (Supplementary Figure 3C), while there was a significant increase in late-stage apoptotic cells (i.e., AnnexinV^+^/7-AAD^+^) (Supplementary Figure 3D). In previous studies, we (17) and others (18) identified that the pro-apoptotic protein BIM (encoded by *Bcl2l11*) is strongly upregulated in NCOR1-deficient thymocytes. A similar upregulation of BIM was also observed in *in vitro*-activated NCOR1 cKO^Cd4^ CD4^+^ T cells compared to *in vitro*-activated WT CD4^+^ T cells (Supplementary Figures 3E,F). However, the *in vitro*-activated NCOR1 cKO^Cd4^ CD4^+^ T cells that were viable displayed increased percentages of IL-2, TNFα, and IFNγ-expressing cells (Figures 2F,G). Further IL-2, TNFα, and IFNγ cytokine expression levels were enhanced compared to *in vitro*-activated WT CD4^+^ T cells, indicating a regulatory role for NCOR1 in controlling cytokine expression in activated CD4^+^ T cells (Figure 2H).

To assess whether the increase in cytokine expression is linked with changes in cell proliferation in the absence of NCOR1, and to test whether proliferating and/or non-proliferating cells display reduced survival, naïve WT and NCOR1 cKO^Cd4^ CD4^+^ T cells were labeled with cell proliferation dye and activated with anti-CD3ε/anti-CD28. Overall, the fraction of cells that divided at least once was significantly higher in WT cells compared to NCOR1-deficient CD4^+^ T cells (Supplementary Figures 4A,B). Further, the percentage of surviving CD4^+^ T cells was higher among the cells that proliferated in comparison to cells that have not proliferated, and the viability of WT CD4^+^ T cells was in both cases higher than the viability of NCOR1 cKO^Cd4^ CD4^+^ T cells (Supplementary Figures 4C–F; upper panels). Nevertheless, the percentage of IFNγ-expressing cells was significantly increased and there was also a tendency that the percentage of IL-2 and TNFα-expressing cells is increased among the alive and dividing NCOR1-deficient CD4^+^ T cells compared to WT CD4^+^ T cells (Supplementary Figures 4C,D). Together, these data demonstrate that NCOR1 is essential for the survival of *in vitro*-activated CD4^+^ T cells and that NCOR1 regulates the percentage of cytokine-expressing cells as well as cytokine expression levels.

### Nuclear Receptor Interaction Domains of NCOR1 Are Dispensable for Survival of Activated CD4^+^ T Cells

NCOR1 mediates ligand-independent transcriptional repression of nuclear receptors (6). The binding of NCOR1 to unliganded nuclear receptors is mediated by three C-terminal receptor interaction domains (RID1-3) (36). To test whether NCOR1 requires nuclear receptor binding for promoting survival of *in vitro*-activated CD4^+^ T cells, we analyzed mice in which NCOR1 conditionally lacks RID2 and RID3 in T cells (NCOR1 RID cKO^Cd4^) and thus does not interact with certain nuclear receptors (14, 37). Lymphoblast formation (Figures 2I,J) and viability (Figures 2K,L) of *in vitro*-activated NCOR1 RID cKO^Cd4^ CD4^+^ T cells was comparable to WT CD4^+^ T cells. Further, the late activation marker CD44 was similarly upregulated in *in vitro*-activated WT and NCOR1 RID cKO^Cd4^ CD4^+^ T cells after 72 h (Figure 2M). Thus, the ability of NCOR1 to interact with nuclear receptors via RID2 and RID3 is not essential for promoting CD4^+^ T cell viability upon activation.

### IFNγ Expression Is Enhanced in NCOR1-Deficient Th1 Cells

**Figure 3:**
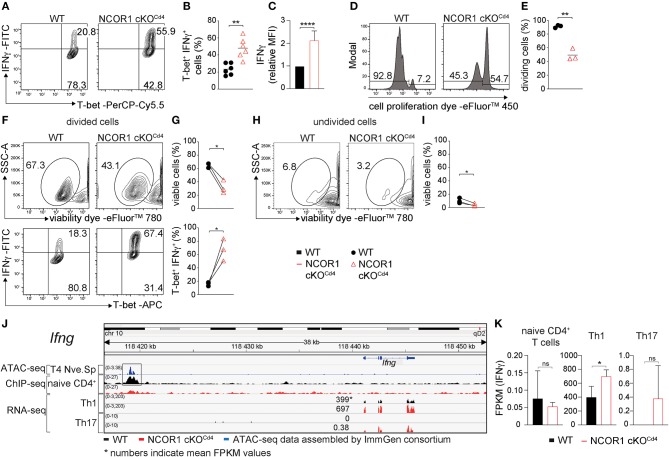
Enhanced IFNγ expression in NCOR1-deficient Th1 cells. **(A)** Naïve WT and NCOR1 cKO^Cd4^ CD4^+^ T cells were activated with anti-CD3ε/anti-CD28 for 3 days in the presence of Th1-inducing cytokines. Contour plots depict T-bet and IFNγ expression. **(B)** Summary of all experiments performed as described in **(A)**. **(C)** Summary graph of mean fluorescence intensity of IFNγ expression levels. For each experiment, WT levels were set as 1 and relative IFNγ levels were calculated. **(D)** Representative histograms depict proliferation. Regions mark divided (left) and undivided cells (right). **(E)** Diagram showing the percentages of cells that underwent at least one cell division. **(F)** Contour plots in the upper panel show viability dye vs. side scatter (SSC-A) for WT and NCOR1 cKO^Cd4^ Th1 cells with at least one cell division (left region in **D**). The lower contour plot panel depicts T-bet and IFNγ expression within proliferated WT and NCOR1 cKO^Cd4^ Th1 cells. **(G)** Summary shows percentages of viable cells (upper graph) and percentages of T-bet^+^IFNγ^+^ cells (lower graph). **(H)** Contour plots show viability dye vs. side scatter (SSC-A) for WT and NCOR1 cKO^Cd4^ Th1 cells of undivided cells (right region in **D**). **(I)** Summary shows percentages of viable undivided cells. **(J)** Integrative Genomics Viewer (IGV) (34, 35) screen shot displaying the *Ifng* gene locus. ImmGen consortium ATAC-seq peaks (GSE 100738) of splenic naïve CD4^+^ T cells are shown in the 1st row. The 2nd and 3^rd^ rows display ChIP-seq tracks from WT and NCOR1 cKO^Cd4^ naïve CD4^+^ T cells, respectively. Rows 4–7 display RNA-seq tracks from *in vitro*-generated WT and NCOR1 cKO^Cd4^ Th1 and Th17 cells. **(K)** Diagrams show the summary of *Ifng* FPKM values obtained by RNA sequencing of naïve CD4^+^ T cells, and of *in vitro*-generated Th1 and Th17 cells. **(B,E)** The horizontal bars indicate the mean. *P < 0.05, **P < 0.01, ****P < 0.0001, ns, not significant (unpaired two-tailed *t*-test or paired *t*-test for **G,I**). **(G,I)** Lines indicate paired experiments. **(A,D,F,H)** Numbers indicate the percentage of cells in the respective quadrants or regions. Data are representative **(A,D,F,H)** or show summary of 6 **(B,C)** and 3 **(E,G,I,K)** samples that were analyzed in 6 **(B,C)** and 3 **(E,G,I,K)** independent experiments.

Next we studied whether NCOR1 functions as a key factor controlling signature cytokine and transcription factor expression, proliferation, and cell viability in differentiated Th1 and Th17 cells. We observed an increase in the percentage of IFNγ-expressing cells as well as an increase in IFNγ expression levels in NCOR1 cKO^Cd4^ CD4^+^ T cells activated under Th1-polarizing conditions, while T-bet expression was not changed in the absence of NCOR1 (Figures 3A–C). Further, the fraction of NCOR1 cKO^Cd4^ Th1 cells that proliferated was reduced compared to WT Th1 cells (Figures 3D,E). There was also diminished viability of proliferating and non-proliferating NCOR1-deficient Th1 cells in comparison to their WT Th1 cell counterparts (Figures 3F–I; upper panel), while the percentage of IFNγ-expressing cells was enhanced in proliferating NCOR1 cKO^Cd4^ Th1 cells (Figures 3F,G; lower panel). In addition, our ChIP-seq experiments indicated NCOR1 binding in naïve CD4^+^ T cells to a region approx. 22 kb upstream of the *Ifng* promoter region (Figure 3J). This region, known as CNS-22, represents a conserved distal regulatory sequence essential for IFNγ expression (38, 39). A search in the ImmGen ATAC-seq database (40) further revealed an open chromatin region in naïve CD4^+^ T cells overlapping with the NCOR1 binding site (Figure 3J). Together with the observation that *Ifng* expression is also enhanced in NCOR1-deficient Th1 cells at RNA level (Figure 3K, Supplementary Table 3), these data suggest that *Ifng* is a NCOR1 target gene and that NCOR1 controls the extent of IFNγ expression in Th1 cells.

In contrast to Th1 cells, WT and NCOR1 cKO^Cd4^ Th17 cells displayed similar percentages of IL-17A and RORγt-expressing cells (Supplementary Figures 5A,B), although IL-17A expression levels were slightly enhanced in the absence of NCOR1 (Supplementary Figure 5C). The fraction of cells that proliferated at least once was slightly reduced in NCOR1 cKO^Cd4^ Th17 cells compared to WT Th17 cells (Supplementary Figures 5D,E), however there was no difference in the viability between WT and NCOR1 cKO^Cd4^ proliferating or non-proliferating Th17 cells (Supplementary Figures 5F–I, upper panel). Further the percentage of IL-17A-expressing cells was similar in WT and NCOR1 cKO^Cd4^ divided Th17 cells. This data indicate that Th17-polarizing cytokines restore cell viability of NCOR1-deficient activated CD4^+^ T cells. Of note, NCOR1 cKO^Cd4^ Th17 cells displayed only a very weak induction of *Ifng* expression compared to WT Th17 cells (Figures 3J,K), which did not express IFNγ at all. This indicates that NCOR1 does not restrain *Ifng* expression in Th17 cells.

### NCOR1 Is a Key Regulator of Transcriptional Landscapes in Th1 and Th17 Cells

To expand the characterization of NCOR1-deficient Th1 and Th17 cells to a genome-wide level, we determined their NCOR1-dependent transcriptomes using RNA-seq approaches. NCOR1-deficient Th1 cells differentially expressed 4,540 genes (2,406 down- and 2,134 upregulated; FDR ≤ 0.05) (Supplementary Figure 6A, left volcano plot), while in NCOR1-deficient Th17 cells there were 5,436 genes dysregulated (2,872 down- and 2,564 upregulated; FDR ≤ 0.05) (Supplementary Figure 6A, right volcano plot). A principal component analysis (PCA) comparing WT and NCOR1-deficient naïve CD4^+^ T cells, Th1, and Th17 transcriptomes indicated grouping of particular Th subsets between WT and NCOR1-deficient CD4^+^ T cells (Supplementary Figure 6B). This suggests that overall Th subset-specific transcriptional programs are maintained in the absence of NCOR1. A detailed analysis of key Th effector genes revealed an upregulation of cytokine genes including *Il2, Il10*, and *Il22* (Supplementary Figures 7A,B), and an induction of cytokine receptors such as *Il7r* and *Ildr1* (Supplementary Figures 7C,D) as well as chemokine receptor genes such as *Ccr1, Ccr2, Ccr3 Ccr4, Ccr5, Ccr8, Cxcr5*, and *Cxcr6* (Supplementary Figures 7E,F) in NCOR1-deficient Th1 and Th17 cells (Supplementary Figures 7A–F, Supplementary Table 3). In contrast, cytokine receptors such as *Ifnar1, Il17ra*, and *Il3ra* (Supplementary Figures 7C,D) and chemokine receptors such as *Ccr7, Cxcr1*, and *Ccrl2* were downregulated in NCOR1-deficient Th1 and Th17 cells (Supplementary Figures 7C–F, Supplementary Table 3). Further, GSEA of Th1 and Th17 RNA-seq datasets revealed an enrichment of hallmark gene sets in NCOR1-deficient Th1 cells such as “Kras signaling up,” “P53 pathway,” “TGFβ signaling,” “Myc targets v2,” and “IL-2 STAT5 signaling,” while in NCOR1-deficient Th17 cells “E2F targets,” “Myc targets v1,” “Myc targets v2,” “G2M checkpoint,” and “oxidative phosphorylation” hallmark gene sets were enriched (Supplementary Figure 6C). The gene set “Myc targets v2” was enriched in both NCOR1 cKO^Cd4^ Th1 and Th17 subsets. These data indicate that NCOR1 is a key regulator of transcriptional landscapes in Th1 and Th17 cells. Of note, *Ncor1* is expressed at highest levels in naïve CD4^+^ T cells followed by lower expression levels in Th17 and Th1 cells (Supplementary Figure 7G, Supplementary Table 3).

Next, we aimed to specifically identify NCOR1-regulated Th1 and Th17 lineage genes, as well as genes that are regulated by NCOR1 only during T cell activation and not during lineage specification. We first defined gene lists specific for WT Th1 cells (i.e., by comparing RNA-seq data from WT Th1 vs. WT naïve CD4^+^ T cells) and WT Th17 cells (WT Th17 vs. WT naïve CD4^+^ T cells) (Supplementary Figure 8). This revealed 10,880 Th1 genes and 10,468 Th17 genes, either Th1/17 lineage-specific or altered due to T cell activation. In a subsequent step, by comparing these Th1 and Th17 gene lists, we identified 2,264 Th1 lineage-specific genes and 1,856 Th17 lineage-specific genes. Further, 8,609 genes were differentially expressed both in Th1 and Th17 cells compared to naïve CD4^+^ T cells and hence designated as “activation-dependent” genes. Subsequently, the lineage-specific or activation-dependent gene lists were compared with the list of genes differentially expressed between WT and NCOR1-deficient Th1 or Th17 cells. This revealed a dysregulation of 666 Th1 lineage-specific genes and 3,030 activation-dependent genes in Th1 cells in the absence of NCOR1, while in NCOR1 cKO^Cd4^ Th17 cells 498 Th17 lineage-specific genes and 3,855 activation-dependent genes were dysregulated. Finally, the overlay of NCOR1 ChIP-seq peaks (naïve CD4^+^ T cells) with the dysregulated genes from the previous analysis revealed 31 (17 up-, 14 downregulated) potential Th1 lineage-specific NCOR1 target genes and 12 (5 up-, 7 downregulated) Th17 lineage-specific NCOR1 target genes. The majority of NCOR1 target genes were activation-dependent genes, with 194 genes (101 up-, 93 downregulated) dysregulated in Th1 cells and 248 genes (76 up-, 172 downregulated) dysregulated in Th17 cells. This indicates that NCOR1 target genes are rather related to overall T cell activation and less to Th1/Th17 lineage specification (Supplementary Table 1).

### Altered Cytokine Expression of NCOR1-Deficient CD4^+^ T Cells *in vivo*

**Figure 4:**
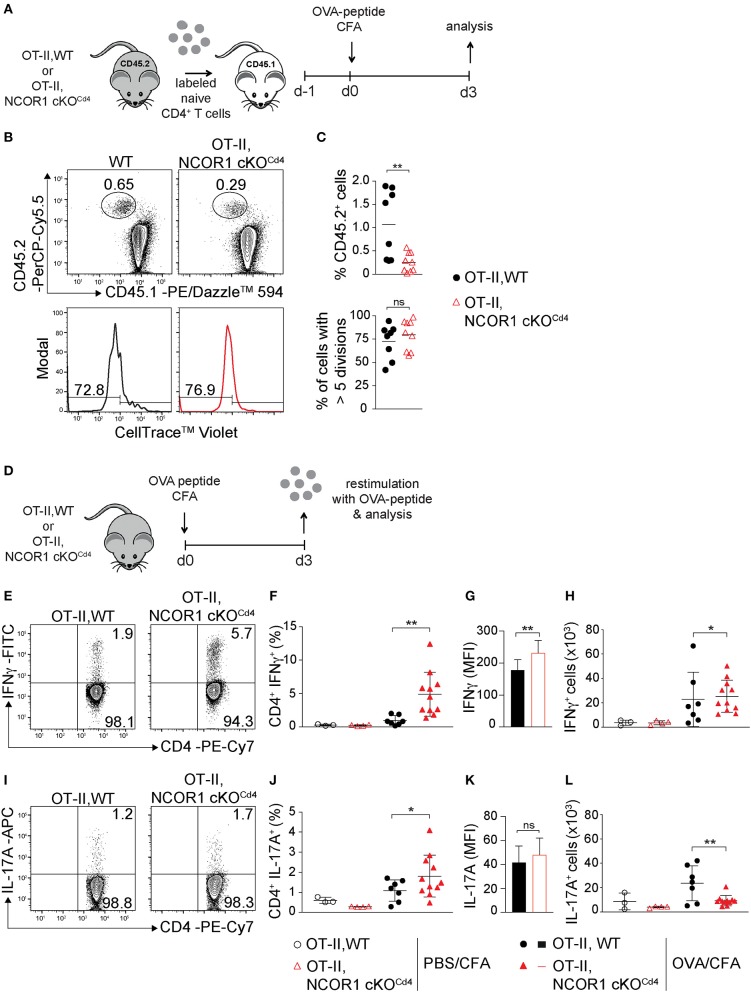
IFNγ and IL-17A expression in NCOR1-deficient CD4^+^ T cells is increased upon *in vivo* short-term activation. **(A)** Experimental set up: Naïve OT-II,WT or OT-II,NCOR1 cKO^Cd4^ CD4^+^ T cells (CD45.2^+^) were labeled with cell tracker dye and transferred into CD45.1^+^ recipient mice (day −1). Mice were immunized s.c. with OVA-peptide/CFA (day 0) and draining lymph node (dLN) cells were analyzed on day 3. **(B)** Contour plots (upper panel) show CD45.1^+^CD45.2^−^ and CD45.1^−^CD45.2^+^ subsets in dLNs. Histograms (lower panel) depict cell proliferation dye dilution of transferred CD45.2^+^ T cells. Left region indicates cells that underwent >5 cell divisions. **(C)** Summary of all experiments performed as described in **(B)**. Upper graph shows the percentage of CD45.2^+^ cells in the dLN (day 3). Lower graph shows the percentage of CD45.2^+^ cells that divided more than 5 times (day 3). **(D)** Experimental set up: OT-II,WT and OT-II,NCOR1 cKO^Cd4^ mice were immunized s.c. with OVA-peptide/CFA. 3 days later dLNs were restimulated with OVA-peptide and analyzed. **(E)** Contour plots depict IFNγ production of restimulated CD4^+^ T cells isolated from OT-II,WT and OT-II,NCOR1 cKO^Cd4^ mice that have been treated as described in **(D)**. **(F)** Summary graph shows the percentage of IFNγ-producing CD4^+^ T cells in dLNs after immunization with OVA-peptide or PBS/CFA control on day 3. **(G)** Summary graph depicts mean fluorescence intensity (MFI) expression levels of IFNγ. **(H)** Summary showing cells numbers of IFNγ^+^ CD4^+^ T cells. **(I)** Contour plots depict IL-17A expression in restimulated OT-II,WT and OT-II,NCOR1 cKO^Cd4^ CD4^+^ T cells. **(J)** Summary graph shows the percentage of IL-17A-producing CD4^+^ T cells in dLNs after immunization with OVA-peptide or PBS/CFA control (day 3). **(K)** Summary graph depicts mean fluorescence intensity (MFI) expression levels of IL-17A. **(L)** Summary showing cell numbers of IL-17A^+^ CD4^+^ T cells. **(C,F,H,J,L)** The horizontal bars indicate the mean. *P < 0.05, **P < 0.01, ns, not significant (unpaired two-tailed *t*-test or ordinary one-way ANOVA for **F,H,J,L**). **(B,E,I)** Numbers indicate the percentage of cells in the respective quadrants or regions. Data are representative **(B,E,I)** or show summary **(C,F–H,J–L)** of 8–9 **(C)** and 3–11 **(F–H,J–L)** mice that were analyzed in 3 independent experiments.

To study the impact of NCOR1 deletion on CD4^+^ T cell proliferation, expansion, and cytokine expression *in vivo*, we crossed WT and NCOR1 cKO^Cd4^ mice with OT-II TCR transgenic mice, which express a TCR restricted to MHC class II (19). MACS-purified CD45.2^+^ OT-II, WT and CD45.2^+^ OT-II, NCOR1 cKO^Cd4^ CD4^+^ T cells were transferred into recipient CD45.1^+^ mice followed by immunization with OVA-peptide/CFA and subsequent assessment of cell proliferation in the draining lymph node (dLN) of recipient mice (Figure 4A). The percentages of CD45.2^+^ OT-II, NCOR1 cKO^Cd4^ CD4^+^ T cells were reduced compared to CD45.2^+^ OT-II, WT CD4^+^ T cells (Figures 4B,C, upper panel). However, transferred WT and NCOR1-deficient CD45.2^+^ OT-II CD4^+^ T cells proliferated at a similar degree (Figures 4B,C, lower panel), suggesting impaired survival of transferred NCOR1-deficient CD4^+^ T cells upon activation. To assess IFNγ expression upon activation *in vivo*, we immunized OT-II, WT and OT-II, NCOR1 cKO^Cd4^ mice with OVA-peptide/CFA or PBS/CFA as a control. After 3 days, dLN cells were isolated and restimulated with OVA-peptide (Figure 4D). Intracellular cytokine staining revealed a strong increase in the percentage of IFNγ-producing CD4^+^ T cells in OT-II, NCOR1 cKO^Cd4^ mice (Figures 4E,F) as well as IFNγ expression levels (Figure 4G), similar to *in vitro*-generated NCOR1-deficient Th1 cells (Figures 3A–C). We also observed a slight increase in the number of IFNγ-expressing cells (Figure 4H). In addition, there was also an increase in the percentage of IL-17A-expressing OTII, NCOR1 cKO^Cd4^ CD4^+^ T cells compared to OTII, WT CD4^+^ T cells (Figures 4I,J), while IL-17A expression levels were not changed (Figure 4K). Of note, absolute cell numbers of IL-17A-expressing cells were reduced in the absence of NCOR1 (Figure 4L). Together, these data indicate that loss of NCOR1 affects both IFNγ and IL-17A expression *in vivo*.

### Attenuated Colitis Induction by NCOR1-Deficient Naïve CD4^+^ T Cells

**Figure 5:**
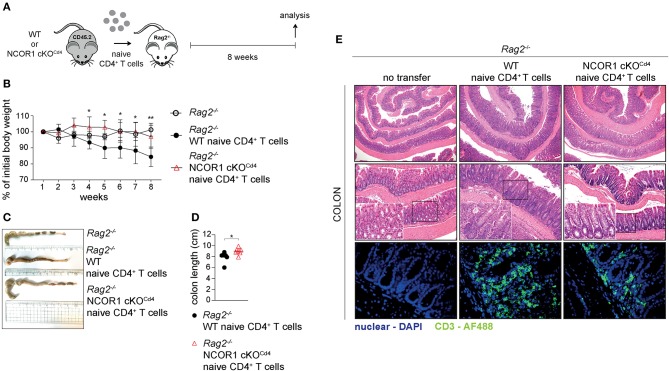
Attenuated induction of colitis by NCOR1-deficient CD4^+^ T cells. **(A)** Experimental set up: Naïve WT or NCOR1 cKO^Cd4^ CD4^+^ T cells were i.p. injected into *Rag2*^−/−^ mice. Eight weeks later, recipient mice were immunophenotyped and analyzed for signs of colitis. Control *Rag2*^−/−^ groups did not receive any cells. **(B)** Diagram showing body weight during the course of the experiment. Body weight at the indicated time point was calculated relative to the initial body weight. **(C)** Representative pictures show colon length of *Rag2*^−/−^ control mice and of *Rag2*^−/−^ mice that have received either naïve WT or NCOR1 cKO^Cd4^ CD4^+^ T cells. **(D)** Diagram shows colon length of *Rag2*^−/−^ mice that have received either naïve WT or NCOR1 cKO^Cd4^ CD4^+^ T cells. Length was measured without cecum. **(E)** Colon swiss rolls were processed for H&E stainings (upper and middle panel). The white rectangle framed pictures in the lower left corner of the middle panel pictures show a 4x magnification of the black rectangle framed section. The lower panel shows immunofluorescence stainings. Color code: blue, nuclei; green, CD3^+^ T cells. Magnification: H&E stainings (upper panel: 40x; middle panel: 100x; and 400x for zoomed in sections) and immunofluorescence stainings (lower panel: 400x). **(D)** Horizontal bar indicates the mean. *P < 0.05, **P < 0.01 (unpaired two-tailed *t*-test). Data are representative **(C,E)** or show summary **(B,D)** of 7–8 **(B–E)** mice that were analyzed in 2–3 independent experiments.

**Figure 6:**
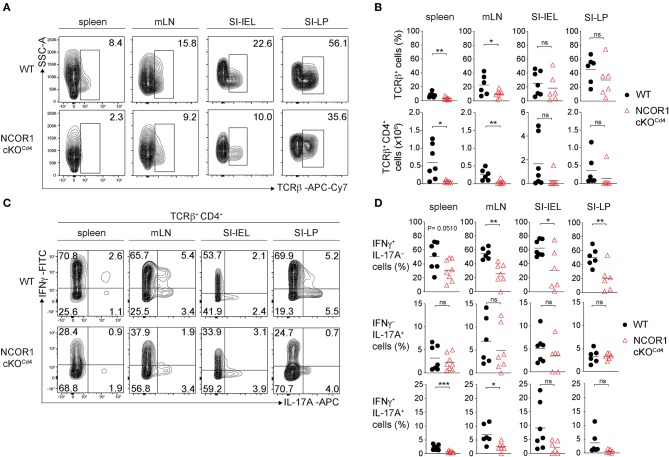
Homeostasis and cytokine expression of NCOR1-deficient CD4^+^ T cells upon transfer. **(A)** Naïve WT or NCOR1 cKO^Cd4^ CD4^+^ T cells were i.p. injected into *Rag2*^−/−^ mice and 8 weeks later transferred CD4^+^ T cells in recipient mice were analyzed. Contour plots show TCRβ expression vs. side scatter (SSC-A) of cells isolated from spleen, mLNs, small intestine IELs (SI-IELs) and lamina propria cells (SI-LPs) of recipient *Rag2*^−/−^ mice. **(B)** Diagrams depict the percentages of TCRβ^+^ cells (upper panel) and numbers of TCRβ^+^ CD4^+^ cells (lower panel) in the indicated organs of *Rag2*^−/−^ mice that have received either naïve WT or NCOR1 cKO^Cd4^ CD4^+^ T cells. **(C)** Contour plots depict IL-17A vs. IFNγ expression of cells isolated from spleen, mLNs, SI-IELs, and SI-LP cells of recipient *Rag2*^−/−^ mice. **(D)** Diagrams indicating percentages of IFNγ single-producing cells (IFNγ^+^IL-17A^−^) (upper panel), IL-17A single-producing cells (IFNγ^−^IL-17A^+^) (middle panel) and IFNγ^+^IL-17A^+^ double-producing (lower panel) TCRβ^+^ cells isolated from *Rag2*^−/−^ mice that have received either naïve WT or NCOR1 cKO^Cd4^ CD4^+^ T cells. **(A,C)** Representative plots for the various organs and tissues were taken from different experiments, however for each organ/tissue shown WT and NCOR1 cKO^Cd4^ plots are from the same experiment. Numbers indicate the percentage of cells in the respective quadrants or regions. **(B,D)** Horizontal bars indicate the mean. *P < 0.05, **P < 0.01, ***P < 0.001, ns, not significant (unpaired two-tailed *t*-test). Data are representative **(A,C)** or show summary **(B,D)** of 6–8 mice that were analyzed in 3 independent experiments.

Next, we investigated the impact of NCOR1 deletion on CD4^+^ T cell-mediated autoinflammation. Due to reduced numbers of peripheral T cells in NCOR1 cKO^Cd4^ mice (17), we employed an adoptive CD4^+^ T cell transfer model of colitis to start with comparable numbers of WT and NCOR1 cKO^Cd4^ CD4^+^ T cells. In this model, the disease is accompanied by body weight reduction, colon length shortening, infiltration of immune cells, and loss of crypt structure in recipient mice (41). Naïve WT and NCOR1 cKO^Cd4^ CD4^+^ T cells were transferred into *Rag2*^−/−^ hosts (Figure 5A) and body weight was monitored over a period of 8 weeks. While *Rag2*^−/−^ mice receiving WT CD4^+^ T cells displayed a body weight loss of approx. 20% after 8 weeks, the transfer of NCOR1-deficient CD4^+^ T cells did not induce weight loss in *Rag2*^−/−^ mice over the 8 week period, similar to control *Rag2*^−/−^ mice that did not receive any CD4^+^ T cells (Figure 5B). Furthermore, colon length of *Rag2*^−/−^ recipients that received WT CD4^+^ T cells was significantly shorter than the colon length of *Rag2*^−/−^ mice receiving NCOR1-deficient CD4^+^ T cells (Figures 5C,D). A histological assessment including an immunofluorescence analysis of the colon after 8 weeks showed massive infiltration of T cells and loss of crypt structure in *Rag2*^−/−^ mice receiving WT CD4^+^ T cells. In contrast, the colon of *Rag2*^−/−^ mice that received NCOR1 cKO^Cd4^ CD4^+^ T cells was less inflamed (Figure 5E). An analysis of the transferred CD4^+^ T cells in *Rag2*^−/−^ recipient mice revealed a tendency (although statistically not significant) of reduced percentages and numbers of small intestine NCOR1 cKO^Cd4^ TCRβ^+^ CD4^+^ IELs (SI-IEL) and LP cells (SI-LP) in comparison to *Rag2*^−/−^ recipients receiving WT CD4^+^ T cells. In contrast, there was a significant reduction of the percentages and numbers of transferred NCOR1 cKO^Cd4^ TCRβ^+^ CD4^+^ T cells in the spleen and mLNs of recipient mice in comparison to transferred WT TCRβ^+^ CD4^+^ T cells (Figures 6A,B). This suggests a tissue-specific effect on the maintenance of NCOR1 cKO^Cd4^ TCRβ^+^ CD4^+^ T cells upon transfer. Moreover, *ex vivo* stimulation of T cells isolated from various tissues and assessment of IFNγ and IL-17A expression revealed that IFNγ-producing cells (IFNγ^+^IL-17A^−^) as well as IFNγ and IL-17A-double producers (IFNγ^+^IL-17A^+^) were reduced in the absence of NCOR1, while the percentage of IL-17A-expressing cells (IFNγ^−^IL-17A^+^) was not changed (Figures 6C,D). Of note, IL-10-producing cells were reduced within NCOR1-deficient SI-IELs and SI-LP cells (Supplementary Figures 9C,D). This indicates that NCOR1 controls cytokine expression in effector CD4^+^ T cell subsets.

## Discussion

In this study we demonstrate a novel role for NCOR1 in the regulation of CD4^+^ T cell homeostasis and effector functions. The first findings of our analysis revealed that NCOR1 is essential for regulating transcriptional landscapes in naïve CD4^+^ T cells as well as in Th1 and Th17 subsets and led to the identification of NCOR1 target genes. NCOR1-deficiency resulted in more than 2,000 differentially expressed genes in naïve CD4^+^ T cells and more than 4,500 genes in Th1/Th17 cells, respectively. NCOR1 binding regions in naïve CD4^+^ T cells mapped to 1,042 gene loci. Approximately one third of NCOR1 binding peaks map to the promoter region of target genes, however a large fraction of potential target genes displayed NCOR1 recruitment within introns and intergenic regions. This suggests that NCOR1 might also control the activity of other *cis*-regulatory elements at target gene loci, such as enhancers and transcriptional silencers. Furthermore, NCOR1 has been shown to function as a transcriptional corepressor by connecting transcription factors with repressive chromatin-modifying enzymes of the histone deacetylase (HDAC) family, in particular HDAC3 (42–45). Due to the recruitment of HDACs, one would have expected far more NCOR1 target genes up- than downregulated in NCOR1-deficient CD4^+^ T cells. Surprisingly, the number of up- and downregulated target genes was similar in the absence of NCOR1, suggesting that NCOR1 might also promote transcriptional activation and thus gene expression.

The transcriptional changes in naïve CD4^+^ T cells caused by the absence of NCOR1 led to the dysregulation of pathways and hallmark gene sets associated with various cellular processes. NCOR1, via binding to LXR, has been described as a key regulator for repressing cholesterol homeostasis-related genes in hepatocytes (46). The observation that cholesterol homeostasis gene sets are enriched in NCOR1-deficient CD4^+^ T cells suggests that NCOR1 has also a similar repressive function in naïve CD4^+^ T cells. Of note, a pathway analysis revealed that the top five canonical pathways upregulated in NCOR1-deficient naïve CD4^+^ T cells displayed an overrepresentation of cholesterol biosynthesis pathways (Supplementary Figure 2B), further supporting the conclusion that NCOR1 regulates cholesterol metabolism in naïve CD4^+^ T cells. Upon CD4^+^ T cell activation cholesterol biosynthesis is increased and its metabolites are crucial for Th effector differentiation and function (47–49). Cholesterol homeostasis gene sets were not enriched in NCOR1-deficient Th1 or Th17 cells, suggesting a specific role for NCOR1 in naïve CD4^+^ T cells in setting up transcriptional programs required for cholesterol metabolism. However, we did not observe binding of NCOR1 to the gene loci of dysregulated members of the cholesterol gene set in naïve CD4^+^ T cells, suggesting either indirect effects or that the NCOR1 ChIP-seq antibody did not recognize NCOR1 in densely packed transcriptional complexes that regulate cholesterol pathway genes. Additional hallmark gene sets enriched (based on NES) in the absence of NCOR1 were “MTORC1 signaling,” “G2M checkpoint regulation,” “Myc targets,” and “E2F targets.” Alterations in the c-Myc pathway might also explain impaired proliferation of activated CD4^+^ T cells in the absence of NCOR1. Since NCOR1 interacts with many different types of transcription factors (7–9), the large number of dysregulated genes and pathways might indicate that NCOR1 orchestrates and integrates the activity of many transcriptional regulators. A *de novo* motif search within NCOR1 binding regions revealed an overrepresentation of an E-box motif (30). This motif is known as a binding site for key T cell transcription factors including c-Myc (50) and the basic helix-loop-helix factor E2A (51), which interact with NCOR1 in other cell types (52, 53). c-Myc has also been reported to recruit NCOR1 to target genes during somatic cell reprogramming (54). Altogether, these findings suggest that in CD4^+^ T cells E-box binding transcription factors represent an important group of regulators that recruit NCOR1 to target gene loci in CD4^+^ T cells.

In our analysis we also observed that the survival of activated CD4^+^ T cells is impaired in the absence of NCOR1. This is reminiscent of the role of NCOR1 during thymocyte development. Previous studies showed that NCOR1 regulates thymocyte survival during negative and positive selection processes (17, 18), and suggested that NCOR1 is one of the factors that integrates TCR signals with thymocyte survival. Since we observed that NCOR1-mediated survival of activated CD4^+^ T cells was not dependent on its ability to interact with nuclear receptors, members of other transcription factor families are likely to utilize NCOR1 in the control of CD4^+^ T cell viability upon activation. Of note, despite the changes of transcriptional landscapes and the reduced viability of activated CD4^+^ T cells in the absence of NCOR1, the deletion of NCOR1 did not affect survival of *in vitro*-generated Th17 cells, indicating that cytokines driving Th17 differentiation might compensate for the loss of a NCOR1-dependent survival program. Moreover, despite the severe transcriptional changes in naïve NCOR1 cKO^Cd4^ CD4^+^ T cells, the lineage-specific expression of the key Th1 and Th17 transcription factors T-bet and RORγt, respectively, was maintained in the absence of NCOR1. This indicates that signaling pathways driving Th1 and Th17 differentiation *in vitro* were functional in the absence of NCOR1. This conclusion is supported by our observation that only a minority of potential NCOR1 target genes, which we defined as bound by NCOR1 in naïve CD4^+^ T cells and dysregulated in Th1 and/or Th17 cells, were associated with Th1 lineage or Th17 lineage specification and function. This suggests that NCOR1 is rather a general regulator of effector programs in Th cells. Nevertheless, *Ifng* showed up as a Th1 lineage-specific gene regulated by NCOR1, which was supported by our *in vitro* data that NCOR1-deficient Th1 cells contain a higher fraction of IFNγ-expressing cells. Naïve CD4^+^ T cells and Th17 cells did not induce IFNγ expression in the absence of NCOR1, suggesting that NCOR1 acts at the *Ifng* locus specifically in Th1 cells. In addition, we showed that NCOR1 binds to CNS-22, which is a regulatory sequence of the *Ifng* locus essential for IFNγ expression in Th1 cells and NK cells (38, 39). Thus, *Ifng* is a NCOR1 target gene and our data suggest that NCOR1 directly negatively regulates the extent of IFNγ expression. In support of this observation, a similar increase in IFNγ-producing cells was also observed *in vivo* upon short-term activation.

Finally, our study demonstrated that NCOR1 is a key regulator of effector programs in Th1 and Th17 cells. Despite a normal Th1/Th17 differentiation pattern based on T-bet and RORγt expression, loss of NCOR1 resulted in the upregulation of many cytokines, cytokine receptors, chemokines, and chemokine receptors, suggesting NCOR1-mediated control of Th1/Th17 cell effector gene expression programs. A key role for NCOR1 in regulating Th effector functions was further identified *in vivo* in an adoptive CD4^+^ T cell transfer model of colitis. Here we observed a severe reduction in crypt disruption and diminished CD4^+^ T cell infiltration in RAG2-deficient mice receiving NCOR1 cKO^Cd4^ CD4^+^ T cells, and this was accompanied with a reduction of IFNγ^+^ as well as IFNγ^+^IL-17A^+^ effector T cell subsets. Since both T cell subsets are linked with the development of colitis (55, 56), it is likely that the reduction of these subsets is one of the contributing factors preventing T cell-mediated pathology by NCOR1-deficient CD4^+^ T cells. In addition, the percentages and numbers of CD4^+^ T cells in the spleen and mLNs of *Rag2*^−/−^ mice that received adoptively transferred NCOR1-deficient CD4^+^ T cells were lower in comparison to recipients receiving WT CD4^+^ T cells, suggesting some impairment in the survival and/or expansion of transferred NCOR1-deficient CD4^+^ T cells. Therefore, one might argue that an overall lower number of CD4^+^ T cells after transfer leads to a diminished inflammatory response and disease severity. However, a few *Rag2*^−/−^ mice that received WT CD4^+^ T cells showed similar low numbers of CD4^+^ T cells in the spleen and mLNs as *Rag2*^−/−^ mice receiving NCOR1 cKO^Cd4^ CD4^+^ T cells, yet these mice still displayed severe T cell infiltration and crypt damage. This suggests that lower numbers of NCOR1-deficient CD4^+^ T cells upon transfer might not be the primary cause for the absence of inflammation, although we cannot rule out that this, together with a disease-protective cytokine expression pattern, contributes to the attenuated colitis pathology. Of note, the diminished population of IFNγ-expressing cells in the adoptive transfer colitis model was an unexpected observation, since we observed that activated NCOR1 cKO^Cd4^ CD4^+^ T cells produced enhanced levels of IFNγ. It is likely that short-term activation of NCOR1-deficient CD4^+^ T cells leads to an increase in IFNγ expression due to direct regulation of the *Ifng* locus by NCOR1. In contrast, upon adoptive transfer and induction of colitis the subset distribution of NCOR1-deficient CD4^+^ T cells is changed and hence the percentage of IFNγ^+^ Th cells might be reduced. It is therefore tempting to speculate that NCOR1 controls effector CD4^+^ T cell subset differentiation *in vivo*. In support of this hypothesis, we also observed that NCOR1 cKO^Cd4^ mice displayed under homeostatic conditions a similar dramatic decrease in IFNγ^+^ CD4^+^ T cells accompanied with a strong increase in IL-17A^+^ CD4^+^ T cells within the SI-IEL population (Supplementary Figures 9A,B). Additionally, due to the altered chemokine expression patterns in NCOR1-deficient Th1 and Th17 cells, loss of NCOR1 might also differentially affect migration and homing of Th subsets into various organs.

Taken together, our study revealed novel gene regulatory functions for NCOR1 in setting up transcriptional landscapes in CD4^+^ T cells and demonstrated an essential role for NCOR1 in T cell-mediated immunopathology.

## Data Availability Statement

The authors declare that the data supporting the findings of this research are available in the article, the Supplementary Material, or on request from the corresponding author. The ChIP-seq and RNA-seq data have been deposited in the GEO database under accession number GSE138934.

## Ethics Statement

Animal experiments were evaluated by the ethics committees of the Medical University of Vienna and approved by the Austrian Federal Ministry for Education, Science and Research (GZ:BMWF-66.009/0105-WF/II/3b/2014, BMBWF-66.009/0039-V/3b/2019).

## Author Contributions

DH designed the research, planned and performed most of the experiments, analyzed the data, and made figures. VS and CZ performed some of the experiments. LM, PH, RR, and AH assisted with some of the experiments. DW and MA performed cell sorting. MS and TK analyzed the sequencing data. CB supervised the sequencing experiments and associated data analysis. SK, MT, and MF analyzed data. WE designed the research and analyzed the data. DH and WE wrote the manuscript with contributions from all co-authors.

### Conflict of Interest

The authors declare that the research was conducted in the absence of any commercial or financial relationships that could be construed as a potential conflict of interest.
