# Supplementary material for: NCOR1 Orchestrates Transcriptional Landscapes and Effector Functions of CD4+ T Cells
Source: Front Immunol. 2020 Apr 3;11:579. doi: 10.3389/fimmu.2020.00579 (PMC7147518; doi:10.3389/fimmu.2020.00579)
Supplement: Supplementary file 1 [file Data_Sheet_1.pdf]

Supplementary Material

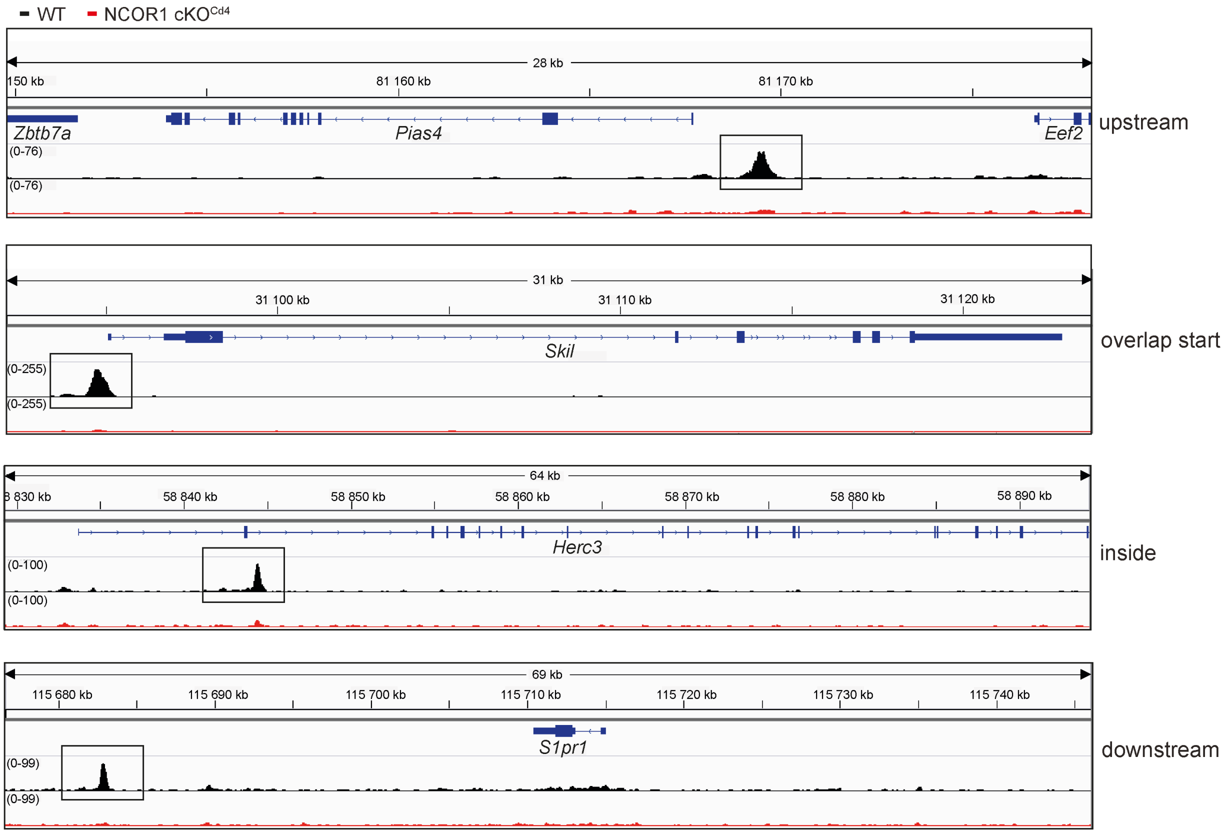

Supplementary Figure 1. ChIP-seq tracks displaying NCOR1 binding peaks at selected gene loci.

Representative Integrative Genomics Viewer (IGV) ChIP-seq coverage tracks of NCOR1 binding peaks in naïve WT CD4^{+} T cells (upper lane) for an upstream binding site (*Pias4*), for a binding site overlapping with the transcriptional start site (*Skil*), for a binding region within the gene body (*Herc3*) and a downstream binding site (*S1pr1*). The lower lane depicts a control ChIP-seq track of NCOR1-deficient naïve CD4^{+} T cells

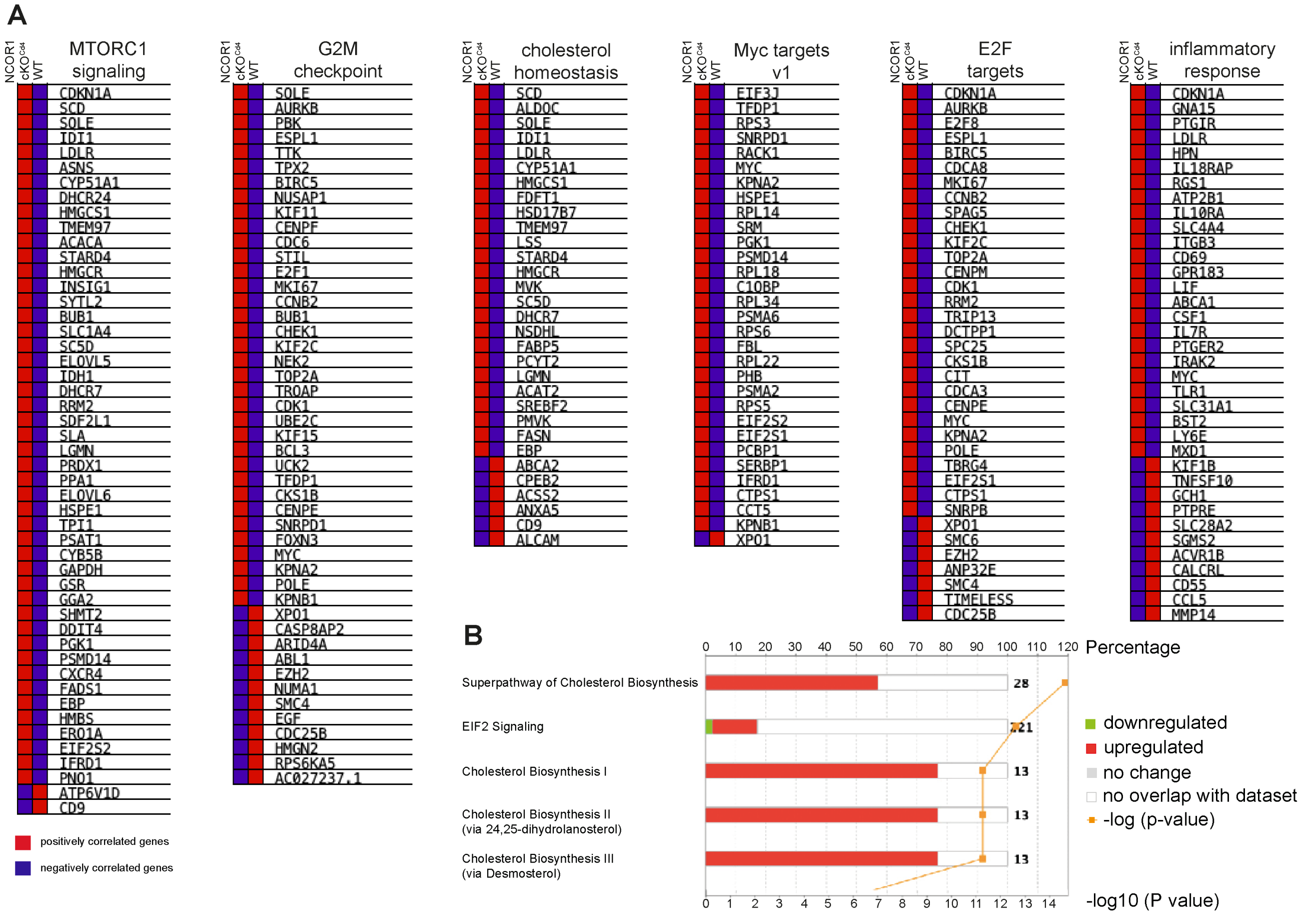

Supplementary Figure 2. Differentially expressed genes of hallmark gene sets enriched in NCOR1-deficient naïve CD4^{+} T cells and upregulation of cholesterol biosynthesis pathways in NCOR1-deficient naïve CD4^{+} T cells.

(A) The top 6 hallmark gene sets enriched in NCOR1-deficient CD4^{+} T cells as revealed by GSEA are shown. Heat maps depict differentially expressed genes of the indicated gene sets. Red squares show positively correlated genes and blue squares negatively correlated genes. (B) Ingenuity Pathway Analysis (IPA) of RNA-seq data from WT and NCOR1 cKO^{Cd4} naïve CD4^{+} T cells. The top 5 canonical pathways are shown. Bars indicate the percentage of genes involved in designated pathways. Numbers at the right indicate the number of genes involved in designated pathways. Orange line indicates P values (–log10). The bar color code: red, upregulated genes; green, downregulated genes; grey, no change and white, no overlap with dataset.

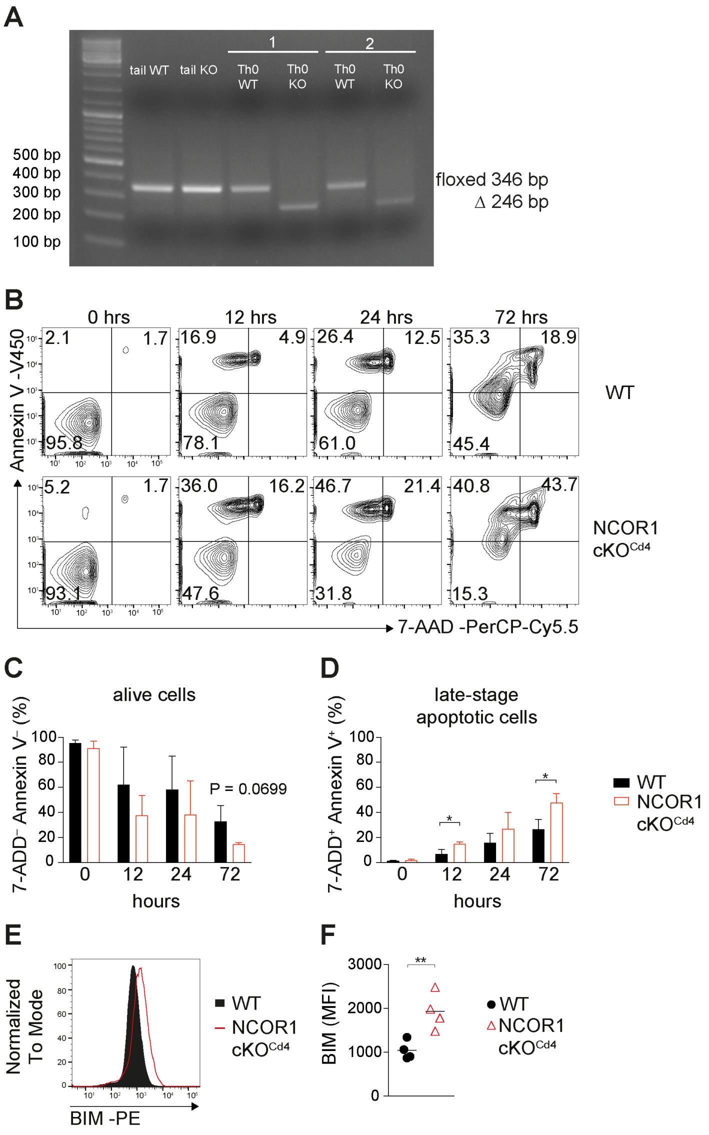

Supplementary Figure 3. Reduced survival of *in vitro*-activated NCOR1-deficient CD4^{+} T cells.

(A) Gel image of semi-quantitative PCR to assess *Ncor1* deletion in *in vitro*-activated CD4^{+} T cells. Lane 1-2: WT and NCOR1 cKO^{Cd4} tail DNA (total tail lysates) used as positive and negative controls, respectively. Lane 3-6: WT and NCOR1 cKO^{Cd4} Th0 cell DNA (total cell lysates) from 2 independent cell batches. (B) Contour plots show 7-AAD versus Annexin V reactivity of *in vitro*-activated WT and NCOR1 cKO^{Cd4} CD4^{+} T cells. (C) Bar chart showing the percentages of alive (7-AAD^{-}Annexin V^{-}) Th0 cells at the indicated time points after activation. (D) Percentages of late-stage apoptotic (7-AAD^{+}AnnexinV^{+}) Th0 cells at indicated time points after activation. (E) Histogram overlay depicts BIM expression in *in vitro*-activated WT and NCOR1 cKO^{Cd4} CD4^{+} T cells. (F) Summary showing BIM mean fluorescence intensity (MFI) expression levels in Th0 cells. (B) Numbers indicate the percentage of cells in the respective quadrants. (F) The horizontal bar indicates the mean. *P<0.05, **P<0.01, (unpaired two-tailed t-test). Data are representative (B,E) or show summary (C,D,F) of 3-4 samples that have been analyzed in 2-3 independent experiments.

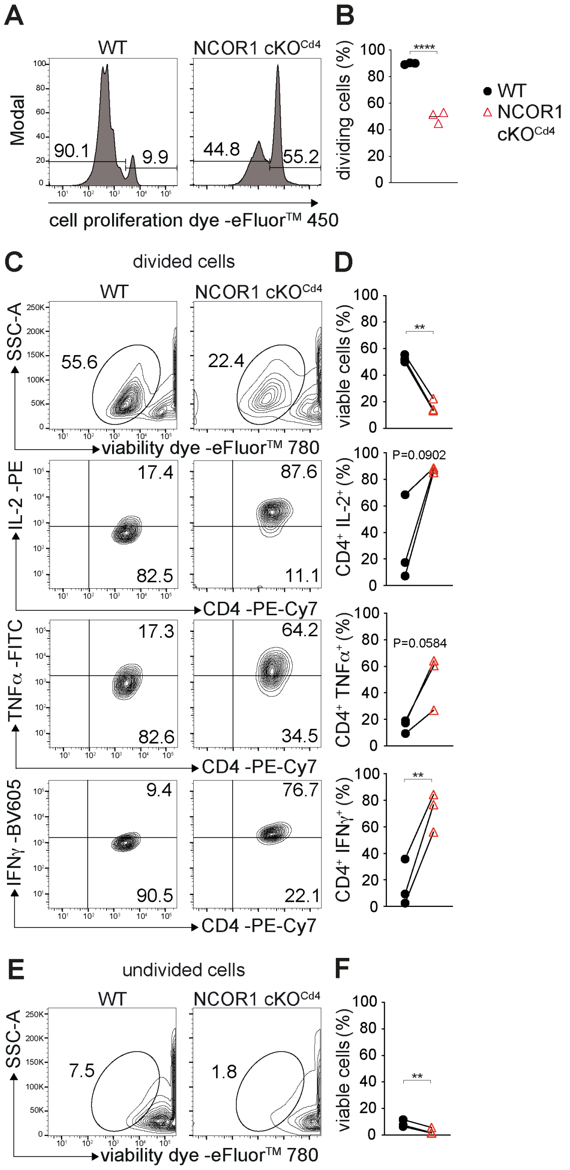

Supplementary Figure 4. Proliferated NCOR1-deficient Th0 cells show increased cytokine production.

(A) Naïve WT and NCOR1 cKO^{Cd4} CD4$^+$ T cells were activated with anti-CD3ε/anti-CD28 and cultured in the presence of recombinant hIL-2 for 3 days (= Th0 condition). Representative histograms depict proliferation. Regions mark proliferated (left) and non-proliferated cells (right). (B) Summary diagram shows the percentages of dividing cells. (C) Contour plots in the upper panel show viability dye versus side scatter (SSC-A) for WT and NCOR1 cKO^{Cd4} Th0 cells with at least one cell division (left region in A). The lower contour plots depict CD4 versus IL-2, TNFα or IFNγ expression. (D) Summary shows percentages of viable cells (upper graph) and percentages of CD4$^+$IL-2$^+$, CD4$^+$TNFα$^+$ and CD4$^+$IFNγ$^+$ cells. (E) and (F) show the same as in the upper panel of C and D, respectively, but of cells that did not proliferate (right region in A). **P<0.01,****P<0.0001, ns, not significant (unpaired two-tailed t-test or paired t-test for D,F). (D,F) Lines indicate paired experiments. (A,C,E) Numbers indicate the percentage of cells in the respective quadrants or regions. Data are representative (A,C,E) or show summary of 3 (B,D,F) samples that were analyzed in 3 independent experiments.

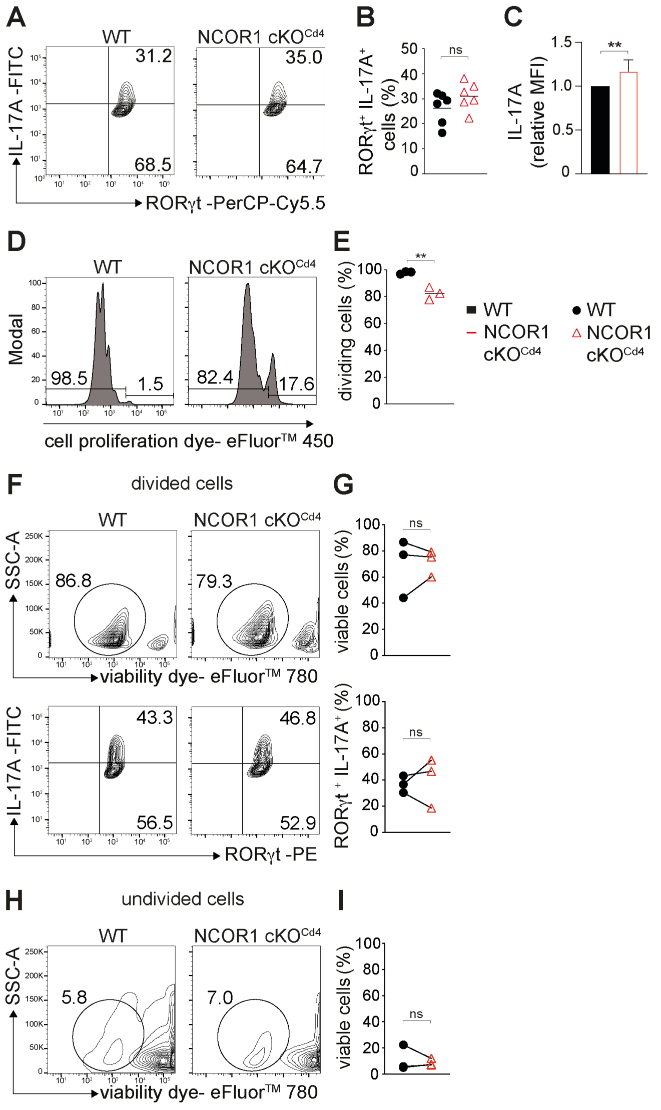

Supplementary Figure 5. Th17-polarizing conditions rescue survival of *in vitro*-activated NCOR1-deficient CD4^{+} T cells.

(A) Naïve WT and NCOR1 cKO^{Cd4} CD4^{+} T cells were activated with anti-CD3ε/anti-CD28 for 3 days in the presence of Th17-inducing cytokines. Contour plots depict RORγt and IL-17A expression. (B) Summary of all experiments performed as described in A. (C) Summary graph of mean fluorescence intensity (MFI) of IL-17A expression levels. For each experiment, WT levels were set as 1 and relative IL-17A levels were calculated. (D) Representative histograms depict proliferation. Regions mark divided (left) and undivided cells (right). (E) Diagram showing the percentages of cells that underwent at least one cell division. (F) Contour plots in the upper panel show viability dye versus side scatter (SSC-A) for WT and NCOR1 cKO^{Cd4} Th17 cells with at least one cell division (left region in D). The lower contour plot panel depicts RORγt and IL-17A expression within proliferated WT and NCOR1 cKO^{Cd4} Th17 cells. (G) Summary shows percentages of viable cells (upper graph) and percentages of RORγt^{+}IL-17A^{+} cells (lower graph). (H) and (I) show the same as in the upper panel of F and G, respectively, but of cells that did not proliferate (right region in D). **P<0.01, ns, not significant (unpaired two-tailed t-test or paired t-test for G,I). (G,I) Lines indicate paired experiments. (A,D,F,H) Numbers indicate the percentage of cells in the respective quadrants or regions. Data are representative (A,D,F,H) or show summary of 6 (B,C) samples that were analyzed in 6 independent experiments or 3 (E,G,I) samples that were analyzed in 3 independent experiments.

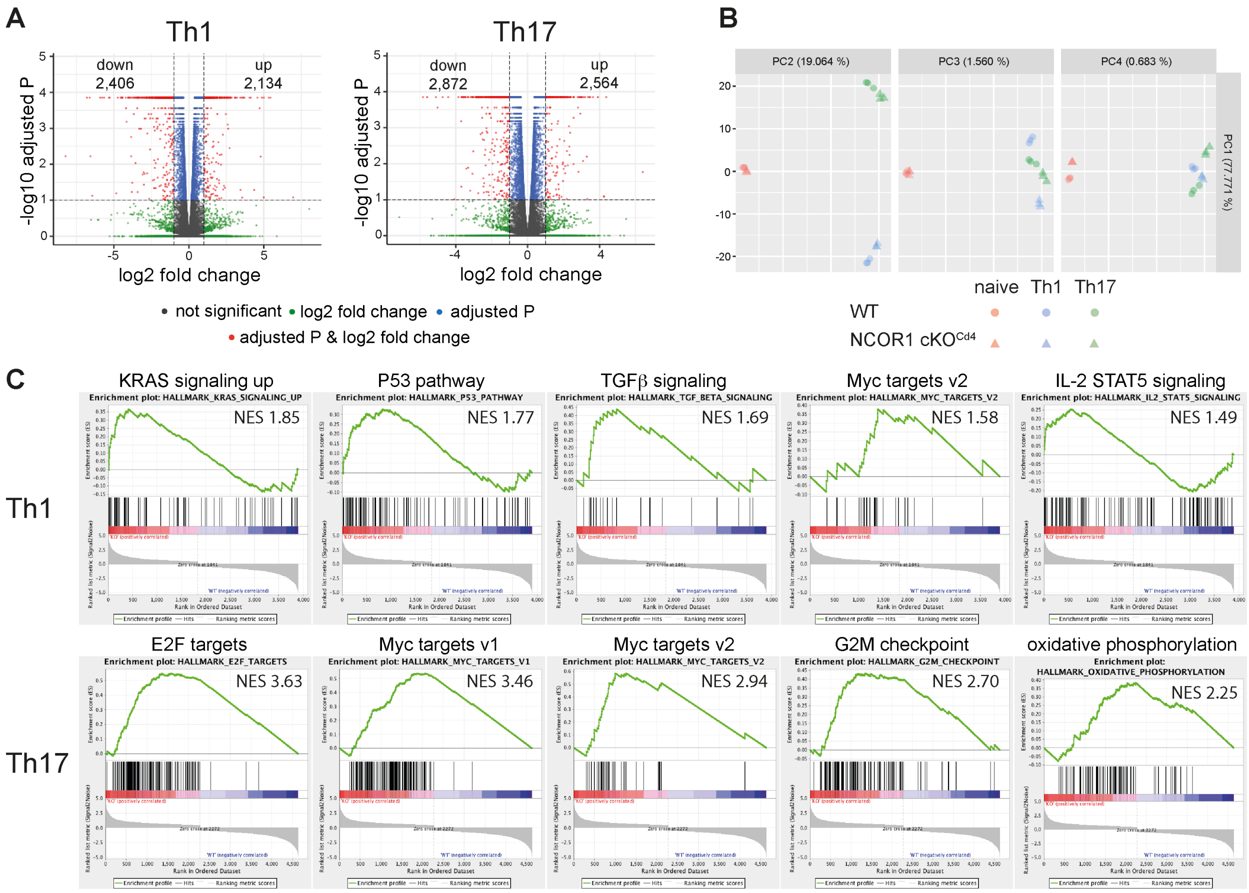

Supplementary Figure 6. Broad dysregulation of gene expression in NCOR1-deficient Th1 and Th17 cells.

(A) RNA isolated from WT and NCOR1 cKO^{Cd4} *in vitro*-generated Th1 and Th17 cells was subjected to RNA sequencing. Volcano plots depict a comparison of global gene expression profiles between WT and NCOR1 cKO^{Cd4} Th1 (left) and Th17 (right) cells. 3 independent batches per genotype were analyzed. The x-axis displays fold change (log2) and the y-axis shows P values (–log10). 2,134 and 2,406 genes were up- and downregulated, respectively, in NCOR1 cKO^{Cd4} Th1 cells, and 2,564 and 2,872 genes in Th17 cells (FDR $\leq$ 0.05 was used as selection criteria). (B) Principal component analysis (PCA)-plot of RNA-seq expression datasets from WT and NCOR1 cKO^{Cd4} naïve CD4^{+} T cells, Th1 and Th17 cells. (C) Gene set enrichment analysis (GSEA) plots with RNA-seq expression data of WT and NCOR1 cKO^{Cd4} Th1 cells (upper panel) and Th17 cells (lower panel). Gene sets of the hallmark gene set collection of the Molecular Signature Database (MSigDB) were used. The top 5 enriched hallmark gene sets are shown. The bar codes indicate the location of the members of the gene set in the ranked list of all genes. NES, normalized enrichment score.

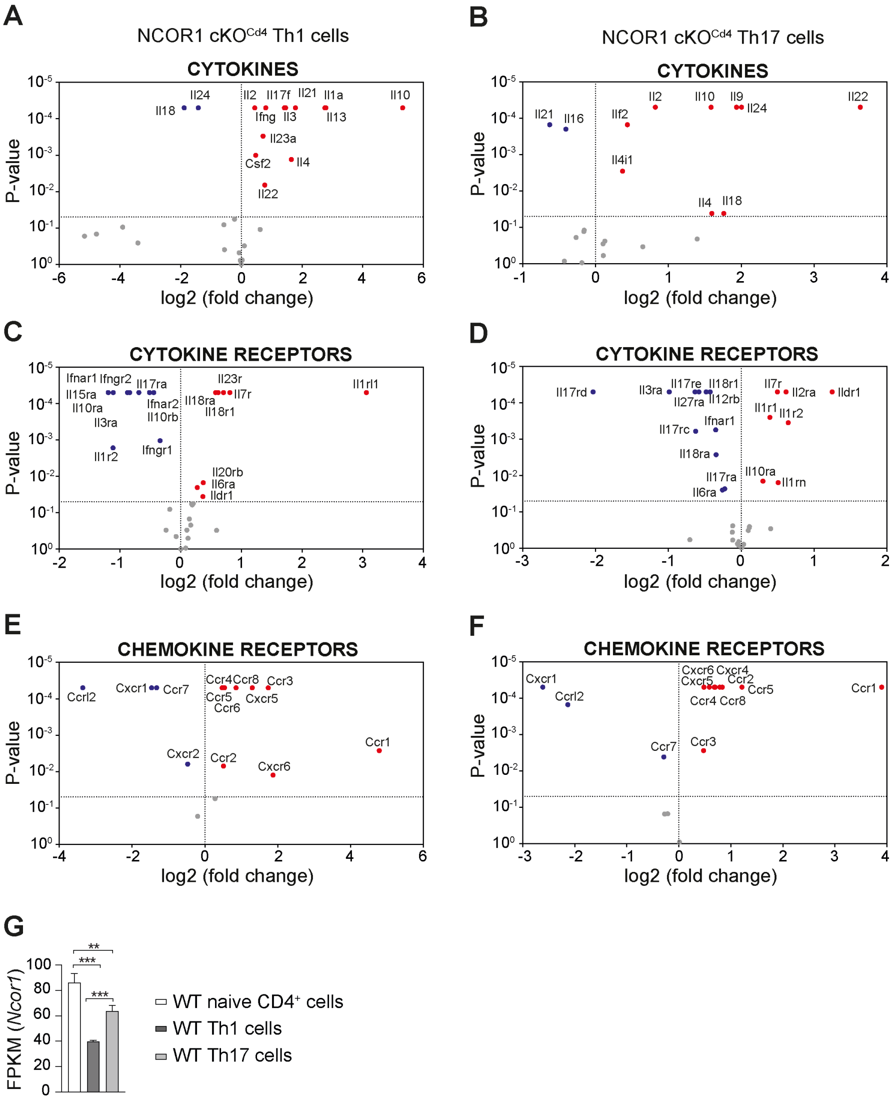

Supplementary Figure 7. Cytokine, cytokine receptor and chemokine receptor expression changes in NCOR1-deficient Th1 and Th17 cells.

(A) RNA from WT and NCOR1 cKO^{Cd4} *in vitro*-generated Th1 and Th17 cells was isolated and subjected to RNA sequencing. 3 independent WT and NCOR1 cKO^{Cd4} CD4^{+} Th1 or Th17 cell batches were prepared on the same day. Volcano plots depict a comparison of cytokine, cytokine receptor and chemokine receptor gene expression profiles between WT and NCOR1 cKO^{Cd4} Th1 cells (A,C,E) and WT and NCOR1 cKO^{Cd4} Th17 cells (B,D,F). The x-axis represents log2 fold change and the y-axis represents P values (FDR $\leq$ 0.05 was used as selection criteria). Red dots indicate upregulated genes and blue dots downregulated genes in NCOR1-deficient cells of the indicated subset. (G) Summary graph depicts FPKM values of *Ncor1* expression in naïve CD4^{+} T cells, *in vitro*-generated Th1 and Th17 cells. $^{**}P<0.01$, $^{***}P<0.001$ (unpaired two-tailed t-test). Data show summary (G) of 3 independent batches for each cell type.

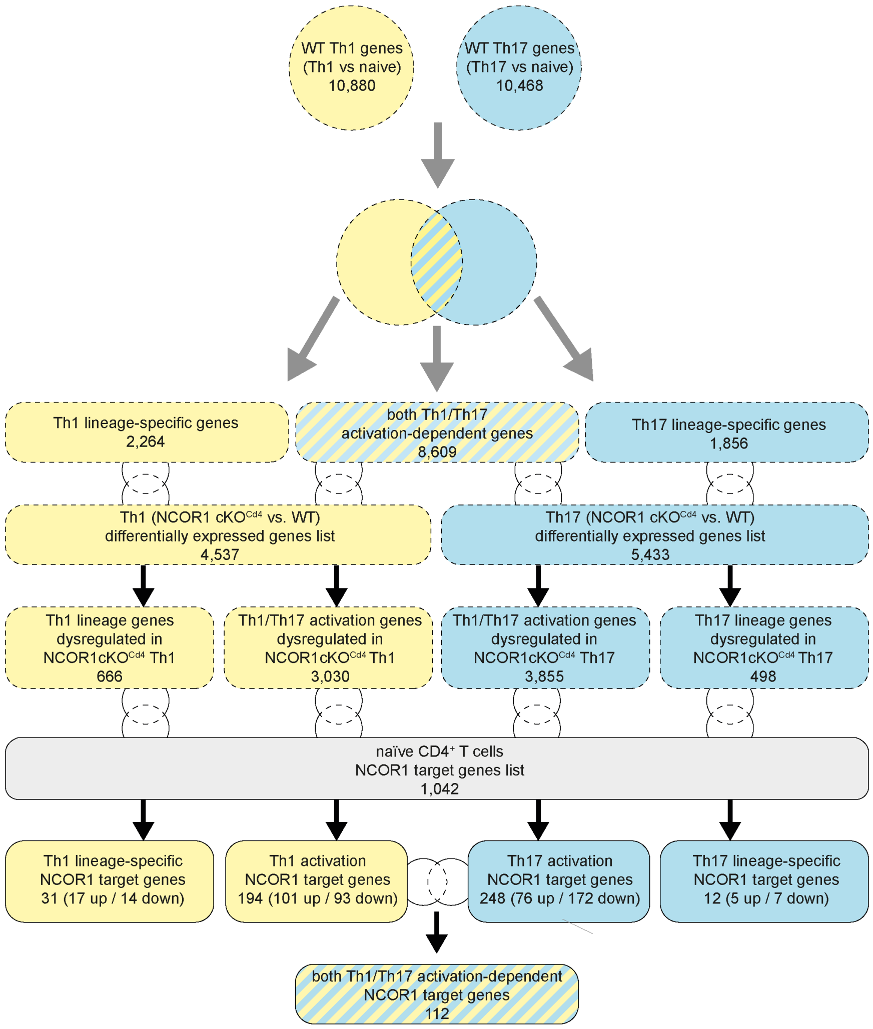

Supplementary Figure 8. Flow diagram showing the analysis of how to identify NCOR1 target genes associated with Th1/Th17 lineage specification or with T cell activation.
Firstly, gene lists specific for WT Th1 cells (i.e. by comparing RNA-seq data from WT Th1 vs WT naïve CD4$^+$ T cells; yellow circle) and WT Th17 cells (WT Th17 vs WT naïve CD4$^+$ T cells; blue circle) were defined. Secondly, Th1 and Th17 gene lists were compared, leading to the identification of Th1 lineage-specific genes (yellow rectangle), Th17 lineage-specific genes (blue rectangle) and "activation-dependent" genes (yellow/blue rectangle). Thirdly, these three gene lists were compared with lists of genes differentially expressed between WT and NCOR1-deficient Th1 or Th17 cells. This revealed Th1 lineage and Th17 lineage-specific genes as well as activation-dependent genes dysregulated in the absence of NCOR1. Finally, the overlay of NCOR1 ChIP-seq peaks (naïve CD4$^+$ T cells) with the dysregulated genes revealed potential NCOR1 target genes in the various categories. Yellow colors indicate comparisons connected with Th1 cells and blue colors indicate comparisons connected with Th17 cells. Dotted lines indicate RNA sequencing data, while solid lines indicate data associated with ChIP sequencing.

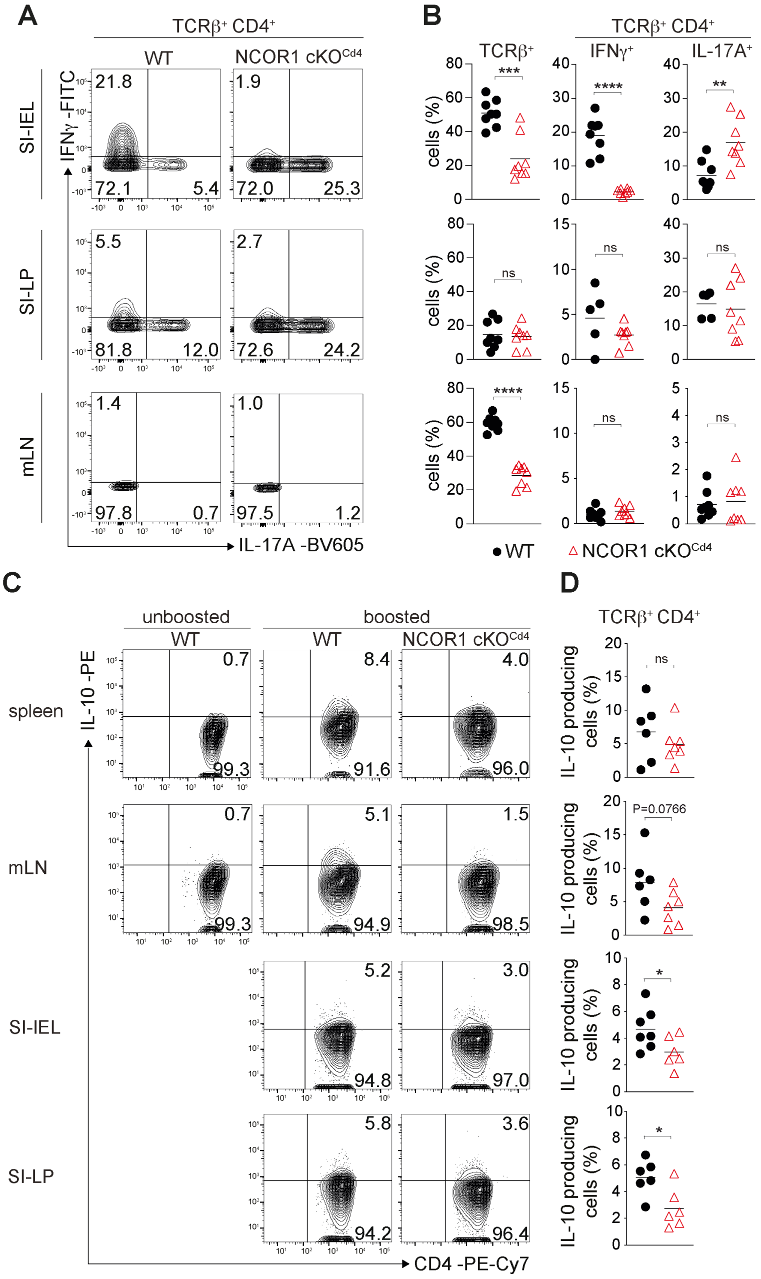

Supplementary Figure 9. IFNγ and IL-17A expression in CD4+ T cells under homeostatic conditions in WT or NCOR1 cKO^{Cd4} mice and reduced IL-10 expression upon transfer.

(A) Contour plots depicting IFNγ and IL-17A expression in small intestine IELs (SI-IEL) (upper panel), in lamina propria cells (SI-LP) (middle panel) and in mLN cells (lower panel) isolated from WT and NCOR1 cKO^{Cd4} mice. (B) Graphs show the percentage of TCRβ$^+$ (left), IFNγ$^+$ (middle) and IL-17A$^+$ cells (right) in the indicated populations of all mice analyzed. (C) Naïve WT or NCOR1 cKO^{Cd4} CD4$^+$ T cells were i.p. injected into *Rag2*$^{-/-}$ mice and 8 weeks later transferred CD4$^+$ T cells in recipient mice were analyzed. Contour plots show CD4 versus IL-10 expression of cells isolated from spleen, mLNs, SI-IELs and SI-LP cells of recipient *Rag2*$^{-/-}$ mice. (D) Diagrams depict the percentages of IL-10-producing cells in the indicated organs (C on the left) of *Rag2*$^{-/-}$ mice that have received either WT or NCOR1 cKO^{Cd4} naïve CD4$^+$ T cells. (A,C) Numbers indicate the percentage of cells in the respective quadrants. (B,D) Horizontal bars indicate the mean. *P<0.05, **P<0.01, ***P<0.001, ****P<0.0001, ns, not significant (unpaired two-tailed t-test). Data are representative (A,C) or show summary (B,D) of 6-8 (except for WT TCRβ$^+$CD4$^+$ LP cells in B, n=5) mice that were analyzed in 2 (B) or 3 (D) independent experiments.

Supplementary Table 1.
Overlaps between ChIP-seq and RNA-seq gene lists reveal NCOR1 target genes.

	NCOR1 target genes										
	naive CD4^{+}		Th1 lineage		Th1 activation		Th17 activation		Th17 lineage		Th1 and Th17 activation
	UP	DOWN	UP	DOWN	UP	DOWN	UP	DOWN	UP	DOWN	UP and DOWN
1	1700056E22Rik	4930417O13Rik	4930509E16Rik	Asf1a	4930417O13Rik	A430078G23Rik	Atf4	2810001G20Rik	Cfl1	Arid5b	4930417O13Rik
2	Art2a-ps	4930453N24Rik	Bbc3	Ccr7	Apol9b	Acp5	BC004004	4930417O13Rik	Chd7	Egr1	A430078G23Rik
3	Art2b	4932438A13Rik	Ddit4	Cd2	Bcl11b	Actn1	Bcl2	4930523C07Rik	Hnrnpdl	Maz	Acp5
4	Bcl2	Abhd8	Hcfc1	D16Ertd472e	Bcor	Adamts14	Bola1	4932438A13Rik	Junb	Mmp9	Actn1
5	Ccdc88b	Adamts14	Hnrnph1	Ifngr1	Birc6	Adamtsl4	Calm2	A430078G23Rik	Olfm2	Nfatc1	Adamtsl4
6	Cd69	Adamtsl4	Ifng	Irgc1	Brd2	Arhgap25	Calm3	Acp5		Sh2d2a	Apol9b
7	Cfl1	Apol9b	Inpp5d	Kctd2	Btg2	Arid5a	Cd69	Actn1		Sirt6	Arhgap25
8	Cited2	Arid1b	Map3k11	Nsmce1	Capg	Art2b	Cdk11b	Adamtsl4			Art2b
9	Cpt1a	Arid5b	Nat10	Nub1	Ccny	Atg16l2	Cpt1a	Ap5z1			Atg16l2
10	Cuedc1	Ash1l	Rasa1	Per1	Cd28	Birc3	Cxcr4	Apol9b			Btg2
11	Cxcr4	B4galnt1	Rasgrp4	Samhd1	Cd4	Cbx4	Cxcr5	Arhgap25			Ccni
12	D16Ertd472e	Bambi-ps1	Rnf145	Stk39	Cd5	Ccni	Cytip	Arhgef1			Cd28
13	Ddit4	Capg	Runx1	Usp3	Cdc42se1	Cd164	Ddx42	Arid1a			Cd4
14	Dph5	Card6	Sbk1	Ypel5	Chd1	Cd72	Dnajc11	Arid1b			Cd72
15	Elovl5	Cbl	Sema4d		Chd3	Cdkn1b	Elovl5	Art2b			Cdkn1b
16	F730043M19Rik	Cd72	Ski		Chrna2	Cnbd2	F730043M19Rik	Atg16l2			Cpt1a
17	Fam71b	Cflar	Skil		Cpt1a	Crem	Fam71b	Atp1b3			Cxcr5
18	Gimap9	Cxcr5			Cxcr5	Ctdsp1	Fnbp1	AW549877			Dirc2
19	Gm10505	Dtx1			Diaph2	Dirc2	Gm27162	Axin2			Dnajc11
20	Gm11529	Dusp2			Dnajc11	E2f2	Gm43672	B4galnt1			Eif2ak3
21	Gm11662	Egr1			Dusp2	Eif2ak3	Grb7	Bambi-ps1			Elf1
22	Gm16085	Egr2			Egr2	Elf1	H2afz	Bin2			F730043M19Rik
23	Gm19585	Entpd5			Eif4a1	F730043M19Rik	Hexim1	Btg2			Fam102a
24	Gm20544	Fam160b1			Eif4a2	Fam102a	Hivep2	Card6			Fam117b
25	Gm25875	Fam3c			Fbxo33	Fam117b	Hmgb1	Ccni			Fli1
26	Gm27162	Fbxo28			Fli1	Gm27162	Hnrnpa2b1	Cd28			Foxo3
27	Gm28935	Foxn2			Foxo3	Gm37357	Hnrnpl	Cd3d			Gm27162
28	Gm29336	Gm8189			Gimap9	Gm37844	Hspa4	Cd4			Gm37844
29	Gm37357	Icos			Gm19585	Gm43769	Hspa5	Cd53			Gm43672
30	Gm43672	Il21r			Gm43672	Gpr146	Hspd1	Cd72			Gpr146
31	Gm43769	Kdm3a			Gpr68	Gramd3	Icos	Cdc42se2			Gpr68
32	Gm5432	Kdm5a			Grb7	Grap2	Il2ra	Cdkn1b			Gramd3
33	Gngt2	Kmt2a			Hdac7	H2afz	Il7r	Cep95			Grb7
34	Grb7	L3mbtl3			Hexim1	Heca	Itk	Cic			H2afz
35	Ifngr1	Lag3			Hist1h1c	Ifi206	Kdm6b	Cuedc1			Heca
36	Il2ra	Ldhd			Hivep2	Jarid2	Klf6	Dennd6b			Hexim1
37	Il7r	Ldlrap1			Hspd1	Kbtbd11	Lbp	Dgkd			Hivep2
38	Itgb7	Lrig1			Hvcn1	Kdm3b	Lrrc8d	Dirc2			Hspd1
39	Izumo1r	Malat1			Il7r	L3mbtl3	Lsm4	Dock2			Hvcn1

	NCOR1 target genes										
	naive CD4^{+}		Th1 lineage		Th1 activation		Th17 activation		Th17 lineage		Th1 and Th17 activation
	UP	DOWN	UP	DOWN	UP	DOWN	UP	DOWN	UP	DOWN	UP and DOWN
40	Lbh	Mcoln2			Kdm6b	Lbp	Med7	Eif2ak3			Il7r
41	Lmna	Mdc1			Klf10	Ldhd	Ncf1	Elf1			Kbtbd11
42	Lrrc6	Mex3b			Klf6	Lockd	Nfkbid	Erbin			Kdm3b
43	Mafg	Mxd4			Kmt2a	Ly96	Nr4a1	Esyt2			Kdm6b
44	Mirt1	Mylip			Lag3	Mcoln2	Osbpl3	Fam102a			Klf6
45	Ncf1	Nfatc1			Laptm5	Mdc1	Pdcd1lg2	Fam117b			Kmt2a
46	Nrip1	Nfia			Lbh	Mitd1	Pdlim4	Fam160b1			L3mbtl3
47	Osgin1	Oplah			Lnpep	Mlec	Pfn1	Fam3c			Lbh
48	Pdcd1lg2	Phip			Lpar5	Mpp7	Plaur	Filip1l			Lbp
49	Pdcd4	Plekhg2			Lrig1	Myl6b	Prdm1	Fli1			Ldhd
50	Pdlim4	Ppp1r16b			Map4k4	Ncf1	Prkag1	Foxo3			Ly96
51	Pgpep1l	Prkd2			Me2	Nr4a1	Prmt5	Gm11707			Map4k4
52	Pim1	Pxylp1			Mex3b	Nr4a3	Prps1	Gm16675			Mex3b
53	Prdm1	Ramp1			Mfng	Nsg2	Ptbp1	Gm20544			Mfng
54	Ptp4a2	Rev3l			Mknk2	Nup210	Ptma	Gm26910			Msra
55	Raly	Rfx3			Msl2	Pag1	Sae1	Gm37844			Myl6b
56	Rasgrp4	Rgcc			Msra	Pdcd4	Sema4a	Gm5711			Ncf1
57	Rhoh	Rnf167			Neurl3	Pdk1	Slc16a6	Gm8189			Nfatc3
58	Rnf145	Scml4			Nfatc3	Pecam1	Smndc1	Gpr146			Nfkbid
59	Rpl27	Sh2b1			Nfkbid	Phip	Snrnp25	Gpr68			Nr4a1
60	Rpl3	Slc41a3			Nfkbiz	Pipox	Snrpc	Gramd3			Nsg2
61	Rpl34	Susd3			Nufip2	Plaur	Socs1	Gramd4			Nup210
62	Rps18	Sypl			Osbpl3	Plcl2	Srrm1	Heca			Osbpl3
63	Samhd1	Tlr6			Pdcd1lg2	Pnrc1	Stk17b	Hist2h2be			Pdcd1lg2
64	Sla	Tmc6			Pitpnc1	Pvr	Tbl1x	Hmg20a			Pdcd4
65	Sntb1	Tnfaip8l2			Prdm1	Pxylp1	Tgfb1	Hvcn1			Pdk1
66	Sorcs2	Tnfrsf14			Prkag1	Rab19	Thy1	Irf1			Pecam1
67	St8sia6	Traf1			Prkd2	Rasa3	Timm9	Itgb7			Plaur
68	Stk17b	Ubash3a			Prmt5	Rbm44	Tk1	Jakmip1			Pnrc1
69	Tbl1x	Wipf2			Pycr2	Rgcc	Tnfrsf10b	Kbtbd11			Prdm1
70	Tgif1	Xpo1			Rap2a	Rmnd1	Tpm3	Kdm2a			Prkag1
71	Tnfrsf10b				Rasal3	Rpl27	Tpm4	Kdm3b			Prkd2
72	Tob1				Rinl	Rpl3	Ube2n	Kdm5a			Prmt5
73	Tox				Sae1	S1pr1	Uspl1	Klf2			Pvr
74	Tpst2				Sfxn3	Scml4	Xpo1	Klf3			Pxylp1
75	Tsc22d3				Sla	Sell	Yy1	Kmt2a			Rab19
76	Zfp239				Smad3	Slfn5	Zc3h12a	L3mbtl3			Rasa3
77					Smad7	Snrnp25		Lbh			Rasal3
78					Sntb1	Sp4		Ldhd			S1pr1

	NCOR1 target genes										
	naive CD4⁺		Th1 lineage		Th1 activation		Th17 activation		Th17 lineage		Th1 and Th17 activation
	UP	DOWN	UP	DOWN	UP	DOWN	UP	DOWN	UP	DOWN	UP and DOWN
79					Spns1	Src		Ldlrap1			Sae1
80					Stk26	St8sia6		Ly96			Scml4
81					Supt4a	Stat3		Malat1			Sell
82					Susd6	Stk10		Map4k4			Sfxn3
83					Tatdn2	Stk17b		Mef2d			Slfn5
84					Tbl1x	Stk4		Mex3b			Smad3
85					Themis	Susd3		Mfng			Snrnp25
86					Timm9	Tdp1		Mir142hg			Sp4
87					Tmc6	Tlr6		Msra			Spns1
88					Tnfrsf10b	Tmem123		Mxd4			St8sia6
89					Tob1	Tnfaip8l2		Myl6b			Stk10
90					Tox	Tpt1		Mylip			Stk17b
91					Trbv31	Tsc22d3		Nfatc3			Stk4
92					Trim8	Ttyh3		Nfia			Tbl1x
93					Trp53inp1	Ubald1		Nipbl			Timm9
94					Ube2n			Nsg2			Tlr6
95					Usp12			Nup210			Tmc6
96					Uspl1			Ogt			Tmem123
97					Vav1			Pan3			Tnfaip8l2
98					Zc3h12a			Pdcd4			Tnfrsf10b
99					Zfp36			Pde3b			Tob1
100					Zfp91			Pdk1			Tox
101					Zswim6			Pecam1			Tpt1
102								Pik3r5			Trbv31
103								Plekhg2			Trim8
104								Pnpla7			Trp53inp1
105								Pnrc1			Tsc22d3
106								Pogz			Ttyh3
107								Prkcq			Ubald1
108								Prkd2			Ube2n
109								Prr12			Uspl1
110								Pten			Zc3h12a
111								Ptpn12			Zfp36
112								Pvr			Zswim6
113								Pxylp1			
114								Rab19			
115								Rac2			
116								Ramp1			

	NCOR1 target genes										
	naive CD4^{+}		Th1 lineage		Th1 activation		Th17 activation		Th17 lineage		Th1 and Th17 activation
	UP	DOWN	UP	DOWN	UP	DOWN	UP	DOWN	UP	DOWN	UP and DOWN
117								Rasa3			
118								Rasal3			
119								Rftn1			
120								Rfx1			
121								Rfx3			
122								Rnf125			
123								Rnf167			
124								Rnf44			
125								S1pr1			
126								Scml4			
127								Sell			
128								Sesn3			
129								Setx			
130								Sfxn3			
131								Sh2d3c			
132								Sh3pxd2a			
133								Slc41a3			
134								Slfn5			
135								Smad3			
136								Sp4			
137								Spns1			
138								Spop			
139								Srrm2			
140								St8sia1			
141								St8sia6			
142								Stk10			
143								Stk4			
144								Tasp1			
145								Tbcel			
146								Tcf7			
147								Tgif1			
148								Tlr6			
149								Tmc6			
150								Tmem123			
151								Tnfaip8l2			
152								Tnfrsf14			
153								Tnik			
154								Tob1			

	NCOR1 target genes										
	naive CD4^{+}		Th1 lineage		Th1 activation		Th17 activation		Th17 lineage		Th1 and Th17 activation
	UP	DOWN	UP	DOWN	UP	DOWN	UP	DOWN	UP	DOWN	UP and DOWN
155								Tox			
156								Tpt1			
157								Trac			
158								Traf1			
159								Traf3ip3			
160								Trbv31			
161								Trim8			
162								Trp53inp1			
163								Tsc22d3			
164								Ttyh3			
165								Txnip			
166								Ubald1			
167								Ubash3a			
168								Vamp1			
169								Zfat			
170								Zfp36			
171								Zfp974			
172								Zswim6			

Supplementary Table 2.
Antibodies and cytokines used in this study.

Antigen	Clone	Fluorochrome or Biotin	Catalog Number	Vendor
CD8α	53-6.7	AF700, V450		BD Biosciences
CD25	PC61	APC		BD Biosciences
CD69	H1.2F3	V450		BD Biosciences
RORγt	Q31-378	PerCPCy5.5, PE		BD Biosciences
Purified NA/LE Hamster Anti-Mouse CD3ε	145-2C11	-		BD Biosciences
Purified NA/LE Hamster Anti-Mouse CD28	37.51	-		BD Biosciences
Bcl6	K112-91	PE-CF594		BD Biosciences
CD4	RM4-5	PE-Cy7		Biolegend
TCRβ	H57-597	APC-Cy7		Biolegend
IL-2	JES6-5H4	PE		Biolegend
TNFα	MP6-XT22	FITC		Biolegend
IFNγ	XMG1.2	APC, FITC, BV605		Biolegend
CD44	IM7	AF700, FITC		Biolegend
T-bet	4B10	PerCP-Cy5.5, APC		Biolegend
IL-17A	TC11-18H10.1	APC, FITC, BV605		Biolegend
IL-10	JES5-16E3	PE		Biolegend
CD45.2	104	PerCP-Cy5.5		Biolegend
CD45.1	A20	PE/Dazzle™ 594		Biolegend
CD62L	MEL-14	PE		Biolegend
Ly-6G/Ly-6C (Gr-1)	RB6-8C5	Biotin		Biolegend
CD45R/B220	RA3-6B2	Biotin		Biolegend
NK1.1	PK136	Biotin		Biolegend
CD11c	N418	Biotin		Biolegend
CD11b	M1/70	Biotin		Biolegend
CD8α	53-6.7	Biotin		Biolegend
TER-119/Erythroid cells	TER-119	Biotin		Biolegend
anti-IL-4	11B11	-		Bioxcell
BIM	C34C5	PE	#2933	Cell Signaling Technology
NCOR1	-	-	#5948S	Cell Signaling Technology
anti-rabbit	N.A	AF488		Invitrogen
anti-CD3 rabbit	SP7	-		Novus

Cytokine	Catalog Number	Vendor
TGFβ	580702	Biolegend
IL-6	575702	Biolegend
IL-1α	211-11a	PeproTech
IL-1β	211-11b	PeproTech
recombinant human IL-2 (rhIL-2)	AF-200-02	PeproTech
IL-12	210-12a	PeproTech

Supplementary Table 3.
Selected RNA-seq FPKM values mentioned in this study.

genes		FPKM values																	
	cell type	Th1			Th1			Th17			Th17			naive $CD4^+$ T cells			naive $CD4^+$ T cells		
	genotype	WT			NCOR1 cKO^{Cd4}			WT			NCOR1 cKO^{Cd4}			WT			NCOR1 cKO^{Cd4}		
	replicates	1	2	3	1	2	3	1	2	3	1	2	3	1	2	3	1	2	3
Ccr1		0.0184574	0.0260664	0.0071164	0.388646	0.603069	0.437632	0.189917	0.180233	0.122648	2.62184	2.16117	2.58281	0.0256748	0.009097	0.0276479	0.0176697	0.0123544	0.0171754
Ccr2		0.68664	0.399727	0.281977	0.517095	0.765502	0.669234	3.73084	3.16612	1.80053	3.82117	5.11977	6.60503	0.216701	0.206704	0.35419	1.23831	1.17999	1.00883
Ccr3		0.0782429	0.0748423	0.0902352	0.204803	0.322357	0.287298	0.752167	0.626417	0.690865	0.844139	0.802729	1.2343	0.0597313	0.0143891	0.0720972	0.0205018	0.0637562	0.0138941
Ccr4		27.1466	30.5037	31.9777	42.99	40.8482	40.8876	33.2374	36.0158	37.887	53.5054	53.1704	54.4454	0.814777	0.629386	0.999116	4.76782	4.22526	4.66446
Ccr5		11.3875	7.51282	6.02439	9.58955	13.3263	13.0075	8.79448	7.90951	5.90604	14.2499	16.6043	21.6264	0.0901009	0.160183	0.195112	0.0872887	0.141354	0.0967596
Ccr7		467.419	497.333	532.43	193.206	195.407	208.539	345.724	326.313	304.228	266.664	264.487	270.778	387.669	290.93	388.584	369.344	328.23	300.043
Ccr8		20.8031	21.2857	26.5403	44.7909	40.3963	38.9801	29.0831	33.6489	48.8114	70.5834	69.9942	51.9763	1.6125	1.67939	1.87619	1.4375	1.6688	1.47681
Ccrl2		5.66717	5.12783	5.72612	0.472714	0.442651	0.70054	0.392712	0.228856	0.405088	0.0693116	0.0363379	0.128614	0.282982	0.372066	0.173451	0.386341	0.173129	0.219935
Cxcr1		3.91123	2.07403	1.06408	0.500974	1.02814	1.02748	10.7515	8.51732	3.42819	0.950728	1.20443	1.55311	1.0876	0.856953	1.35375	0.369934	0.386416	0.226709
Cxcr5		1.7422	1.59063	1.25277	3.25303	3.84488	4.22121	15.9143	15.5605	18.6528	25.0859	23.603	21.4493	2.28031	2.4217	3.38454	2.79159	1.86639	1.54912
Cxcr6		0.34375	0.417099	0.482634	1.61046	1.72585	1.21342	17.8808	19.3393	19.0207	32.4721	29.6184	28.4358	1.02204	1.14543	1.4765	1.01002	1.31443	1.14825
Ifnar1		32.2766	34.1395	33.3202	14.8236	15.744	15.7179	22.1441	20.5764	17.9586	14.6372	15.9062	17.1246	48.9896	53.1019	49.4945	54.826	55.2255	53.4039
Ifng		575.405	350.539	270.754	620.888	804.305	666.598	0	0	0	0.219675	0.912623	0	0.0364135	0.167464	0.0227717	0.0486265	0.0669373	0.0432783
Il10		0.644382	0.391585	0.405476	14.5359	26.5401	16.5074	7.12617	7.60137	5.58898	21.1156	19.2937	20.613	0	0.0208827	0.0212104	0.0390051	0.028957	0.0194489
Il17ra		22.9158	18.4205	17.0746	13.3911	14.0631	13.474	50.0387	49.321	43.8911	39.1731	40.7	42.8202	47.0999	52.2025	43.433	39.3551	42.1043	40.3589
Il2		8.94893	27.1937	35.4112	36.1081	32.5199	28.9586	1.75117	2.48397	1.83951	4.23258	3.17619	3.32465	0.0188911	0.0263246	0.0513624	0.026156	0.0359336	0.0497367
Il22		0.48514	0.358991	0.18554	0.361285	0.855324	0.548723	0.308612	0.288032	0.218995	4.41546	3.32549	2.40628	0	0	0	0	0	0
Il3ra		11.8613	12.6087	10.7496	7.14942	7.49615	7.24011	22.1455	19.4132	16.8815	9.24264	9.67419	10.5686	5.01864	4.37906	3.57183	5.69076	3.90499	4.29431
Il7r		14.9983	11.1774	10.3532	18.6169	22.8691	22.6726	42.177	44.6398	41.6464	45.7834	63.4388	72.3192	177.531	210.983	162.702	240.867	223.05	311.695
Ildr1		0.788871	0.532218	0.399656	0.53511	0.713872	0.969319	2.75954	2.37168	1.75583	5.56054	3.91922	6.90067	1.02286	0.695603	1.00472	2.19802	2.18994	1.86329
Ncor1		40.2543	40.6435	38.878	16.2003	16.4539	16.7446	68.2722	59.4907	63.4654	19.0695	20.76	21.5764	79.0416	93.2456	86.6161	46.4404	46.8484	49.4937
